# The synergism of *SMC1A* cohesin gene silencing and bevacizumab against colorectal cancer

**DOI:** 10.1186/s13046-024-02976-2

**Published:** 2024-02-16

**Authors:** Maddalena Di Nardo, Simonetta Astigiano, Silvia Baldari, Maria Michela Pallotta, Giovanni Porta, Simona Pigozzi, Annalisa Antonini, Laura Emionite, Annalisa Frattini, Roberto Valli, Gabriele Toietta, Silvia Soddu, Antonio Musio

**Affiliations:** 1 Istituto di Tecnologie Biomediche (ITB), Consiglio Nazionale delle Ricerche (CNR), Via Moruzzi, Pisa, 1 56124 Italy; 2 IRCCS Ospedale Policlinico San Martino, Genoa, Italy; 3 Dipartimento Ricerca e Tecnologie Avanzate, IRCCS Istituto Nazionale Tumori Regina Elena, Rome, Italy; 4 Dipartimento di Medicina e Chirurgia, Sezione di Biologia Generale e Genetica Medica, Università degli Studi dell’Insubria, Varese, Italy; 5 Dipartimento di Scienze Chirurgiche e Diagnostiche Integrate, Università degli Studi di Genova, Genoa, Italy; 6 Istituto di Ricerca Genetica e Biomedica (IRGB), Consiglio Nazionale delle Ricerche (CNR), Milan, Italy

**Keywords:** Cohesin, SMC1A, shRNA, Bevacizumab, Colorectal cancer, Gene expression dysregulation

## Abstract

**Background:**

SMC1A is a subunit of the cohesin complex that participates in many DNA- and chromosome-related biological processes. Previous studies have established that *SMC1A* is involved in cancer development and in particular, is overexpressed in chromosomally unstable human colorectal cancer (CRC). This study aimed to investigate whether *SMC1A* could serve as a therapeutic target for CRC.

**Methods:**

At first, we studied the effects of either *SMC1A* overexpression or knockdown in vitro. Next, the outcome of *SMC1A* knocking down (alone or in combination with bevacizumab, a monoclonal antibody against vascular endothelial growth factor) was analyzed in vivo.

**Results:**

We found that *SMC1A* knockdown affects cell proliferation and reduces the ability to grow in anchorage-independent manner. Next, we demonstrated that the silencing of *SMC1A* and the combo treatment were effective in increasing overall survival in a xenograft mouse model. Functional analyses indicated that both treatments lead to atypical mitotic figures and gene expression dysregulation. Differentially expressed genes were implicated in several pathways including gene transcription regulation, cellular proliferation, and other transformation-associated processes.

**Conclusions:**

These results indicate that *SMC1A* silencing, in combination with bevacizumab, can represent a promising therapeutic strategy for human CRC.

**Supplementary Information:**

The online version contains supplementary material available at 10.1186/s13046-024-02976-2.

## Background

Colorectal cancer (CRC) is a global health challenge and its incidence rate is growing worldwide. In accordance with the International Agency for Research on Cancer statistics in 2018, CRC ranked fourth in incidence and third in mortality. There were about 1,096,000 new cases and over 551,000 deaths cases worldwide every year and, in 2021, are estimated about 150,000 new cases in the United States alone [1, 2]. CRC is classified into two main types of carcinogenesis: microsatellite instability (MSI, 15% of patients, associated with a better prognosis) and chromosomal instability (CIN, 85% of cases, with a worse prognosis) [3]. The consequence of CIN is an imbalance in chromosome number with chromosome gain or loss (a phenomenon referred as aneuploidy), genomic amplifications, and a high frequency of loss of heterozygosity (LOH). CRC development requires many years as a consequence of the accumulation of specific mutations in tumor suppressor genes and oncogenes. Inactivation of the *APC* tumor suppressor gene occurs first, followed by activating mutations of *KRAS*. Subsequent cancer progression is driven by additional mutations in the *BRAF*, *PI3K,* and *TP53* genes [4]. Surgery remains the primary choice of CRC though further conventional therapies include chemotherapy, radiotherapy, immunotherapy, and cell therapy, either alone or in combination. However, the survival outcome of patients remains poor, and therapies may lead to severe side effects and emergence of tumor resistance. Thus, there is an urgent need to explore new prognostic biomarkers and therapeutic targets in order to develop better prognosis and more effective precision pharmaceutical treatments for patients with CRC.

The cohesin complex consists of four subunits, SMC1A, SMC3, RAD21, and STAG1/2, forming a ring-shaped structure. It plays key roles in correct chromosome segregation, in gene expression regulation, chromatin remodeling, and DNA repair [5–7]. Somatic variants in the cohesin genes are associated with several types of human cancer including lung carcinoma [8], breast cancer [9, 10], urothelial bladder carcinoma [11–15], glioblastoma [16, 17], Ewing’s sarcoma [18–20], melanoma [21], myeloid neoplasms [22–25], and CRC [26–29]. Since cohesin participates in a growing number of chromatin-related processes, its contribution to cancer development is multifaceted [6].

Among cohesin complex subunits, SMC1A is particularly interesting because it is a target of ATR and ATM kinases and also plays a role in a signal transduction pathway that brings out a checkpoint response to DNA damage for preserving genome stability [30–33]. It has been suggested that *SMC1A* participates in CRC tumorigenesis by promoting aneuploidy [26, 27, 34] and we have previously shown that colorectal tissues acquired extra-copies of *SMC1A* during tumorigenesis and its expression is significantly more robust during cancer progression. It is worth noting that *SMC1A* overexpression has been identified as a predictor of poor prognosis in CRC [28]. In addition, in an experimental model, overexpression of *SMC1A* reduced tumor latency and significantly increased tumor size [29]. These findings might have important clinical applications because *SMC1A* could serve as a potential target for the development of new therapies in CRC. To gain further insight into this matter, we investigated the effect of *SMC1A* knockdown in vitro and in a murine xenograft model. We performed treatments with *SMC1A*-specific shRNA alone or in combination with bevacizumab (Avastin®). Bevacizumab was the first recombinant humanized murine IgG1 monoclonal antibody capable of blocking the activity of the Vascular Endothelial Growth Factor A (VEGF-A), a natural ligand that plays a pivotal role in tumor angiogenesis [35]. In 2004, bevacizumab was approved by the United States Food and Drug Administration (FDA) for the first-line treatment of metastatic CRC [36].

Here, we report that silencing of *SMC1A* in human CRC cells in vitro caused the appearance of abnormal mitotic figures, a significant decrease in cell viability, and decreased capability of anchorage-independent growth. Performing xenotransplant experiments in immunodeficient mice, we found that administration of *SMC1A*-specific shRNA reduces tumor growth and increases the overall survival. Of relevance, survival was even higher when the treatment with shRNA was combined with bevacizumab. The increased mouse survival induced by the combo was associated with high frequency of spontaneous micronuclei levels and abnormal mitotic figures. Finally, gene expression profiles allowed us to identify thousands of dysregulated genes involved in pivotal biological pathways. In conclusion, our work suggests that *SMC1A* (alone and in combination with bevacizumab) represents a potential therapeutic target for human CRC.

## Material and methods

### Cell culture

HCT116, HCT116 overexpressing *SMC1A*, HCT116 knocked down for *SMC1A* by specific shRNA (from heron, HCT116, *SMC1A*-Ov and *SMC1A*-Kd respectively), SW620, and HT29 human cells were grown in Dulbecco’s minimal essential medium (DMEM, Gibco BRL) supplemented with 100 U/ml penicillin, 0.1 mg/ml streptomycin, and 1% L-glutamine in a humidified 5% CO_2_ atmosphere at 37 °C. The generation of *SMC1A*-Ov cells has been described previously [27], while those *SMC1A*-Kd were obtained as reported below. To perform all experiments described in this manuscript, we did not select specific clones but used two different polyclonal cell populations. Cells at early passages following lentiviral vector-mediated transduction were trypsinized (day 0), cultured and tested at different days for cell viability and for the presence of abnormal mitotic figures.

### Lentiviral vector preparation

Bacterial glycerol stocks for a pLKO.1-based lentiviral shRNA plasmid targeting human *SMC1A* (TRCN0000299440), or a non-targeting shRNA plasmid (mock) were obtained from Sigma-Aldrich (St. Louis, MO, USA). The plasmid DNAs were purified using the Plasmid Maxi Kit (Qiagen, Hilden, Germany) and transfected into human embryonic kidney 293 T cells along with a lentiviral packaging plasmid mix to produce lentiviral vectors. Viral-containing supernatants were collected 48 h after transfection, concentrated using the Lenti-X concentrator (Takara Bio Inc., Shiga, Japan) according to the manufacturer’s protocol, titrated by serial dilution in 293 T cells and stored at -80 °C for further use. Cells were transduced with the lentiviral shRNA vector targeting human *SMC1A* in the presence of 6 μg/ml polybrene for 24 h, followed by puromycin selection (1 μg/ml) for 10 days to obtain *SMC1A*-Kd polyclonal cell populations. Two independent infection experiments were performed to obtain two different polyclonal cell populations.

### MTT assay

Parental HCT116 and their derivative polyclonal populations, i.e., *SMC1A*-Ov and *SMC1A*-Kd cells, were seeded into 96-well plates at a concentration of 2 × 10^3^ cells/well. MTT (3-(4,5-dimethyl-thiazol-2-yl)-2,5-diphenyltetrazolium bromide, 5 mg/ml) was added to each well and cells were incubated at 37 °C for 4 h. The formazan crystals produced were dissolved by adding acidified isopropanol (in 0.01 M HCl) to each well and the optical density read on a microplate reader at wavelength of 595 nm.

### Anchorage-independent growth assay

The soft-agar assay was performed as previously described [37]. Briefly, HCT116, *SMC1A*-Ov and *SMC1A*-Kd cells were suspended in 3 ml of 0.3% agar (Difco) supplemented with complete medium. Cell suspension was allowed to solidify at room temperature on 4 ml of a 0.5% agar base layer containing growth medium in 60-mm dishes. After 2 weeks, colonies were stained with crystal violet and counted.

### Animals and experimental design

Thirty-four 5-week-old immunodeficient CD1® nu/nu female mice were purchased from Charles River (Calco, CO, Italy) and housed at the Animal Facility of the IRCCS Ospedale Policlinico San Martino of Genova with 12-h dark/light cycles. Water and food were provided ad libitum. Experimental design was approved by the IRCCS Ospedale Policlinico San Martino of Genova ethics committee (OPBA) and was authorized by the Italian Ministry of Health (Auth 405/2020-PR). All procedures on animals were performed according to the National and European guidelines for care and use of laboratory animals (EEC directive 276/33/2010 and D.L. 26/2014).

Mice were subcutaneously injected in the dorsal right flank with 3 × 10^6^ SMC1A-Ov cells (34 mice) and randomly distributed into four experimental groups. Treatments and tumor measurements were started 4 days later and for 3 consecutive weeks. Weights were recorded once a week for the 3 weeks of treatment.

Treatment groups were as follow: The anti-VEGF group, eight animals received bevacizumab 5 mg/Kg i.p. in 100 μl. One animal did not develop a tumor and was discarded. The shRNA group, nine animals were administered intratumor with lentiviral particles in 20 μl (viral stocks 1.0 × 10^7^ transduction units/ml) once a week. The combo group, nine animals received both bevacizumab and shRNA. Control group, eight animals were given saline solution both i.p. and intratumor.

All animals were monitored daily, and tumors were measured with calipers twice a week by the same operator. End points for sacrifice were 60 days from cell injection or when the tumor volume exceeded 800 mm^3^, a value below that approved by the OPBA and the Ministry of Health (> 1.5 cm^3^). At sacrifice, all animals underwent necropsy, and no metastasis was detected. Tumor samples were collected and anonymized for subsequent analysis; one part of the tissue was snap frozen in liquid nitrogen and one part was fixed in 10% neutral-buffered formalin for at least 48 h and then embedded in paraffin through automatic processing.

### Western blotting

Whole cell protein extracts from HCT116, *SMC1A*-Ov, and *SMC1A*-Kd cells were obtained with lysis buffer [Tris HCl pH 8.0; 25 μM; NaCl 55 μM; EDTA 1 μM; Protease Inhibitor Cocktail (Sigma- Aldrich)] and protein concentration estimated by the Bradford Protein Assay (Thermo Scientific). Proteins, 20 μg per lane, were separated by SDS-PAGE. Proteins were transferred onto nitrocellulose membranes (Amersham) and incubated with anti-SMC1A primary antibody (Fortis Life Sciences). After removal of the unbound primary antibody, membranes were incubated with secondary antibody-peroxidase conjugate (Sigma), processed for detection by chemiluminescence (Amersham) and imaged by Chemidoc (Biorad). Anti-tubulin antibody was used as loading control.

The ImageJ software was used to carry out semiquantitative image analysis of immunoblotting data, expressed by percent of *SMC1A*-Kd (or *SMC1A*-Ov)/control ratio.

### RNA-sequencing (RNA-seq)

Four tumors deriving from the inoculation of *SMC1A*-Ov (14-14 s, 15-15 s, 16-16 s, and 43-48 s), six deriving from *SMC1A*-Ov cells treated with *SMC1A*-specific shRNA (8-8 s, 10-10 s, 11–9-2 s, 22-23 s, 31-32 s, and 52-58 s), eight deriving from *SMC1A*-Ov cells treated with bevacizumab (7-7 s, 14–35-2 s, 16–50-2 s, 26-27 s, 38-43 s, 44-49 s, 46-51 s, and 47-52 s), and eight deriving from *SMC1A*-Ov cells treated with the combo (21-22 s, 27-28 s, 33-34 s, 48-54 s, 49-55 s, 53-59 s, 54-60 s, and 55-61 s) were separately processed for RNA-seq analyses as previously described [38, 39]. Briefly, library preparations were obtained using the TruSeq Stranded mRNA Sample Prep kit (Illumina), starting with 1–2 μg of good quality RNA (R.I.N. > 7) as input. The poly-A mRNAs were fragmented for 3 min at 94 °C and every purification step was performed using 1X Agencourt AMPure XP beads. The quality of both RNA samples and final libraries was tested using the Agilent 2100 Bioanalyzer RNA Nano assay (Agilent). Libraries were then processed with Illumina cBot for cluster generation on the flow cell, following the manufacturer’s instructions and sequenced on single-end mode on HiSeq 2500 (Illumina). The CASAVA 1.8.2 version of the Illumina pipeline was used to process raw data for format conversion and de-multiplexing. For the analysis of differentially expressed genes, the quality-assessed reads were processed using the TopHat version 2.0.0 package (Bowtie 2 version 2.2.0) as FASTQ files. Reads were mapped to the human reference genome GRCh37/hg19. Cuffdiff from the Cufflinks 2.2.0 package was used to calculate the differential expression levels and evaluate the statistical significance of detected alterations. Only protein-coding genes were considered, and gene level expression values were determined by fragments per kilobase million (FPKM) mapped. All genes with FPKM > 1 were designated as expressed and analyzed with an established *p*-value < 0.05.

### Pathway analysis and function

The differentially expressed genes were functionally analyzed for biological processes using Database for Annotation, Visualization, and Integrated Discovery (DAVID) v2023q2 (https://david.ncifcrf.gov). For each term, the p-value was calculated and a term with *p* < 0.05 was considered to be enriched.

### cDNA synthesis and quantitative real-time PCR (qPCR)

Total RNA was extracted by RNAeasy Mini-kit (Qiagen) and cDNA was synthesized with SuperScript™ II reverse transcriptase using oligo-dT (Invitrogen). PCR analyses were performed using Rotor Gene 3000 (Corbett). qPCR reactions were run in triplicate and normalized with respect to HPRT. Primers used for mRNA expression analysis are listed in Supplementary Table S1.

### Immunohistochemistry

Apoptosis was evaluated by immunohistochemistry staining for activated Caspase 3 on 5 μm sections of formalin-fixed/paraffin-embedded tumor samples. We quantified positive areas on seven tumors from control mice (*SMC1A*-Ov); six from shRNA, six from bevacizumab-treated mice; and seven from combo treatment. After hydration and heat-induced antigen retrieval in citrate buffer pH 6, the rabbit Mab A32328 (Bioworld Technology) was diluted 1:100 and incubated for 1 h at RT in 1xTBS [20 mM Tris; 150 mM NaCl pH 7.6] containing 3% BSA and 0.5% Tween 20. Antibody reaction was visualized with MACH 4 Universal HRP polymer detection and Betazoid DAB (both from Bio-Optica). Slides were then digitalized with Aperio eSlide Manager (Leica), visualized with Aperio ImageScope software (Leica). Positive and negative areas were drawn and measured with ImageScope on at least three different randomly chosen fields for each tissue section.

### Immunofluorescence

For immunofluorescence, cells were fixed in 2% paraformaldehyde for 10 min, permeabilized for 5 min on ice in 0.2% Triton X-100 and blocked in PBS with 1% BSA for 30 min at room temperature. Thereafter, cells were incubated with anti-α-tubulin antibody (Abcam) for 1 h, washed in PBS, 1% BSA and incubated with Alexa Fluor 488-conjugated goat anti-rabbit secondary antibody (Molecular Probes) for 1 h. DNA was stained with DAPI. Immunofluorescence experiments were performed in triplicate. Abnormal mitotic figures were evaluated according to parameters described previously [40, 41]. Slides were analyzed using a Leica DM2500 microscope.

### Statistical analyses

All statistical analyses were performed using the SPSS statistical package, version 28.0 (SPSS Inc., USA) for Windows. All data were presented as mean ± standard deviation (SD). Differences between continuous variables were analyzed using the Student’s *t*-test. A *p*-value < 0.05 was considered statistically significant.

## Results

### shRNA-mediated SMC1A knockdown reduces cell proliferation in vitro

**Fig. 1:**
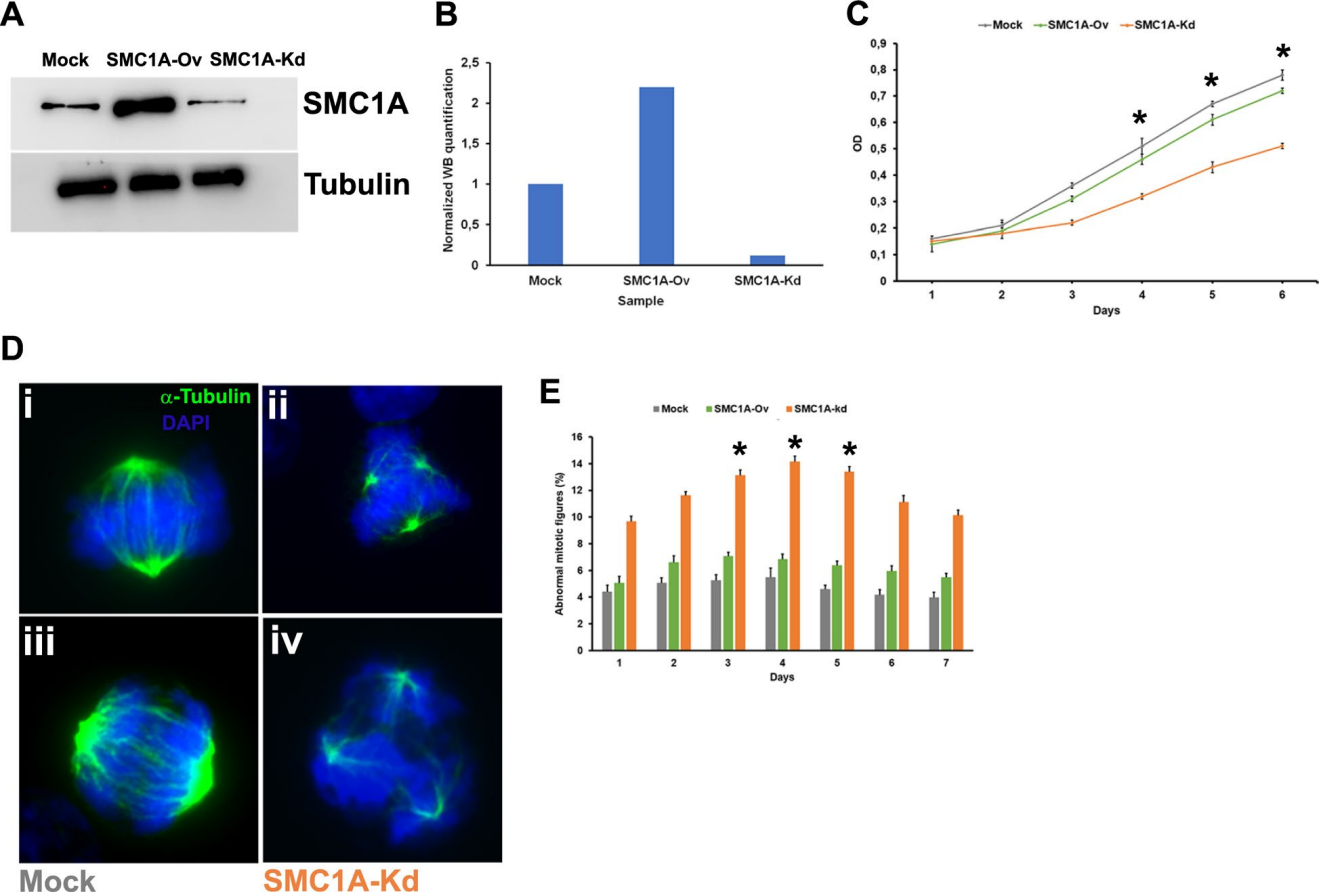
Effects of both *SMC1A* overexpression and silencing in vitro. **A** HCT116 transfected with vectors overexpressing or silencing *SMC1A*. Transfections lead to the overexpression of SMC1A protein or its downregulation when compared to mock cells 24 h after the transfection. Tubulin antibody was used as loading control. **B** ImageJ software was used to carry out the semiquantitative image analysis of immunoblotting data.** C** *SMC1A* silencing affects cell proliferation rate starting from 4th day of in vitro progression. **D** A normal metaphase (i, mock-treated cell), a tripolar metaphase (ii, *SMC1A*-Kd cell), a normal anaphase (iii, mock-treated cell), a tripolar anaphase (iv, *SMC1A*-Kd cell). DNA was stained with DAPI (blue), and mitotic spindle was detected by an anti-α -tubulin antibody (green). **E** Kinetics of abnormal mitotic figures over 7 days. *SMC1A*-kd cells show a peak of atypical figures at 4^th^ day of cell culture. *p < 0.05

At first, we transfected HCT116 cells with two vectors: the first overexpressing *SMC1A* (*SMC1A*-Ov) and the second one containing a *SMC1A-*specific shRNA (*SMC1A*-Kd). Western blot analysis showed that both vectors were effective in inducing, respectively, overexpression of *SMC1A* and its silencing when compared to mock-transfected HCT116 cells (Fig. 1A, Supplementary Fig. 1A). In particular, the *SMC1A*-Ov vector induced more than twice the expression of SMC1A while the *SMC1A*-Kd vector led to a reduction of 90% of SMC1A expression, as analyzed by Image J (Fig. 1B). Thereafter, we investigated the effect on cell viability in vitro. *SMC1A*-Kd cells showed a lower viability rate compared with mock and *SMC1A-*Ov cells. Results of the MTT assay showed a significant reduction of viability in *SMC1A*-Kd starting from day 4 of the cell culture (Fig. 1C). Immunofluorescence staining revealed that *SMC1A*-Kd cells display a significant increase in abnormal figures when compared to *SMC1A*-Ov (*p* = 0.013) and mock (*p* = 0.0028) cells (Supplementary Fig. 1B). Indeed, 12.7% (32 out of 252) of *SMC1A*-Kd mitoses showed altered morphology vs 7% (18 out of 256) and 5.5% (14 out of 255) of mock and SMC*1A*-Ov mitoses, respectively. Figure 1D shows representative images of normal metaphase, with well-organized bipolar mitotic spindles and the chromosomes aligned on the equatorial plate (i); an abnormal tripolar metaphase (ii); a normal anaphase (iii), and a tripolar anaphase (iv). This observation prompted us to analyze the kinetics of abnormal figures occurring over a period of 7 days. Data derived from the analysis of 600 mitoses indicated that *SMC1A*-Kd cells showed an increasing trend of abnormal figures that reached a peak (14% of analyzed cells) after 4 days of cell culture. Afterwards, the number of abnormal mitoses decreases, likely due to apoptotic process (see below), although it always remains higher than mock and *SMC1A*-Ov cells which displayed a uniform trend (Fig. 1E).

Finally, we examined the effect of *SMC1A* knockdown on anchorage-independent growth, a hallmark of cancer cells associated with their tumorigenic potential [42]. To this end, cells were cultured in semisolid agar medium that allows the formation of colonies of transformed cells. Supplementary Table S2 shows the relative plating efficiency (RPE), that is the ratio of plating efficiency in soft agar to plating efficiency in liquid medium. Mock and *SMC1A*-Ov cells successfully grew in semisolid culture forming colonies (RPE = 98.7 and 84.8, respectively), whereas *SMC1A*-Kd cells grew in soft agar with a much lower efficiency (RPE = 49.4%).

Altogether, these results confirm that the silencing of *SMC1A* inhibits both cell proliferation and anchorage-independent growth [28, 43], possibly through induction of reduced cell-fitness due to increased mitotic abnormalities.

### shRNA-mediated SMC1A knockdown in a murine xenograft model

**Fig. 2:**
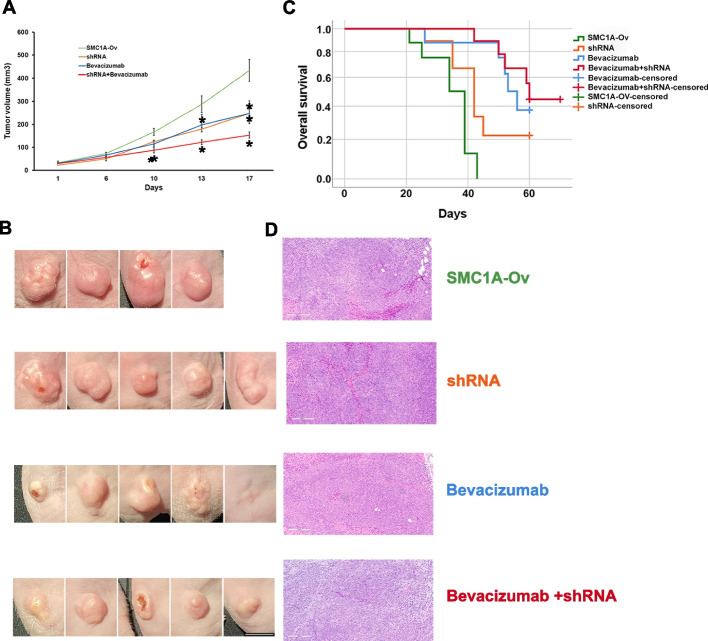
Effects of *SMC1A* silencing and bevacizumab (alone or combined) in vivo. **A** Difference in tumor volume after *SMC1A*-Ov subcutaneous cell inoculation and subsequent treatment with shRNA against SMC1A and bevacizumab (alone or combined). **B** Kaplan–Meier estimates of overall survival. shRNA and bevacizumab improve the life span of mice and this outcome is stronger after combined treatment. **C** Representative images of tumors formed in the mice with *SMC1A*-Ov, shRNA, bevacizumab, and bevacizumab + shRNA treatment. Scale bar: 1 cm. **D** Example of representative histopathological examination performed with hematoxylin and eosin staining. Enlargement 500x. *p < 0.05

In vitro results prompted us to investigate the effects of *SMC1A* knockdown in vivo in immunodeficient CD1® nu/nu mice. Thirty-four female mice were subcutaneously injected in the dorsal right flank with 3 × 10^6^ *SMC1A*-Ov cells and randomly assigned to four different treatment groups: control group (eight mice) received vehicle alone; aVEGF group (eight mice) was treated i.p. with bevacizumab; shRNA group (nine mice) was injected intratumor with *SMC1A-*specific shRNA; combined group (nine mice) received the combo treatment. The time-dependent analysis showed that the volume of the tumors significantly decreased in mice inoculated with *SMC1A-*specific shRNA starting from the 13th day (*p* = 0.04) and in mice treated with bevacizumab after 17 days (*p* = 0.05) when compared with the *SMC1A*-Ov control group. Interestingly, combo treatment significantly reduced the volume of tumors already after 10 days (*p* = 0.04) (Fig. 2A&B). Distributions of time-to-event variables for overall survival were estimated with the Kaplan–Meier product-limit method. This test showed that overall survival was significantly higher in mice treated with shRNA (*p* = 0.04) and bevacizumab (*p* = 0.01). It is worth noting that the difference with the control group was highly significant with combo (*p* = 0.000) (Fig. 2C, Supplementary Table S3). Examples of tumors derived from the four groups are showed in Fig. 2D.

Taken together, these data suggest that *SMC1A* silencing leads to a decrease in tumor volume and an increase in overall survival and these outcomes are improved by the combo treatment, *SMC1A* inhibition plus bevacizumab.

### Effects of both SMC1A silencing and bevacizumab treatment on gene expression in induced tumors

**Fig. 3:**
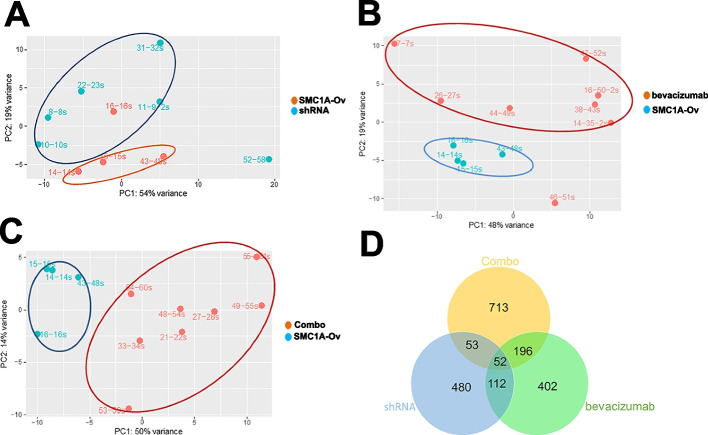
Gene expression profiles in induced tumors. **A** PCA of tumors treated with shRNA. **B** PCA of tumors treated with bevacizumab. **C** PCA of tumors treated with shRNA plus bevacizumab. All tumor spots appeared to be separated from the control spots. **D** Venn diagram of differentially expressed genes following the different treatments. All samples share 52 genes

Next, we obtained gene expression profiles by RNA-seq of 26 tumors, in particular, 4 tumors deriving from the inoculation of *SMC1A*-Ov cells (14-14 s, 15-15 s, 16-16 s, and 43-48 s), 6 deriving from *SMC1A*-Ov cells treated with *SMC1A*-specific shRNA (8-8 s, 10-10 s, 11–9-2 s, 22-23 s, 31-32 s, and 52-58 s), 8 deriving from *SMC1A*-Ov cells treated with bevacizumab (7-7 s, 14–35-2 s, 16–50-2 s, 26-27 s, 38-43 s, 44-49 s, 46-51 s, and 47-52 s) and 8 deriving from *SMC1A*-Ov cells treated with the combo (21-22 s, 27-28 s, 33-34 s, 48-54 s, 49-55 s, 53-59 s, 54-60 s, and 55-61 s). Unsupervised sample clustering by principal component analysis (PCA) clearly differentiated *SMC1A*-Ov samples from shRNA- and bevacizumab-treated samples (Fig. 3A-C). Globally, all samples appeared separated from the control (*SMC1A*-Ov) spot maps, except for SMC*1A*-Ov 16-16 s sample which fell in the shRNA-treated group (Fig. 3A), which in turn were strictly grouped, thus showing a low statistical variance.

Tumors treated by *SMC1A*-specific shRNA and by bevacizumab displayed 697 (442 down and 255 up) and 762 (406 down and 356 up) dysregulated genes, respectively, when compared with *SMC1A*-Ov induced tumors (Fig. 3D, Supplementary Tables S4 & 5, Supplementary Figures S2 & 3).

**Fig. 4:**
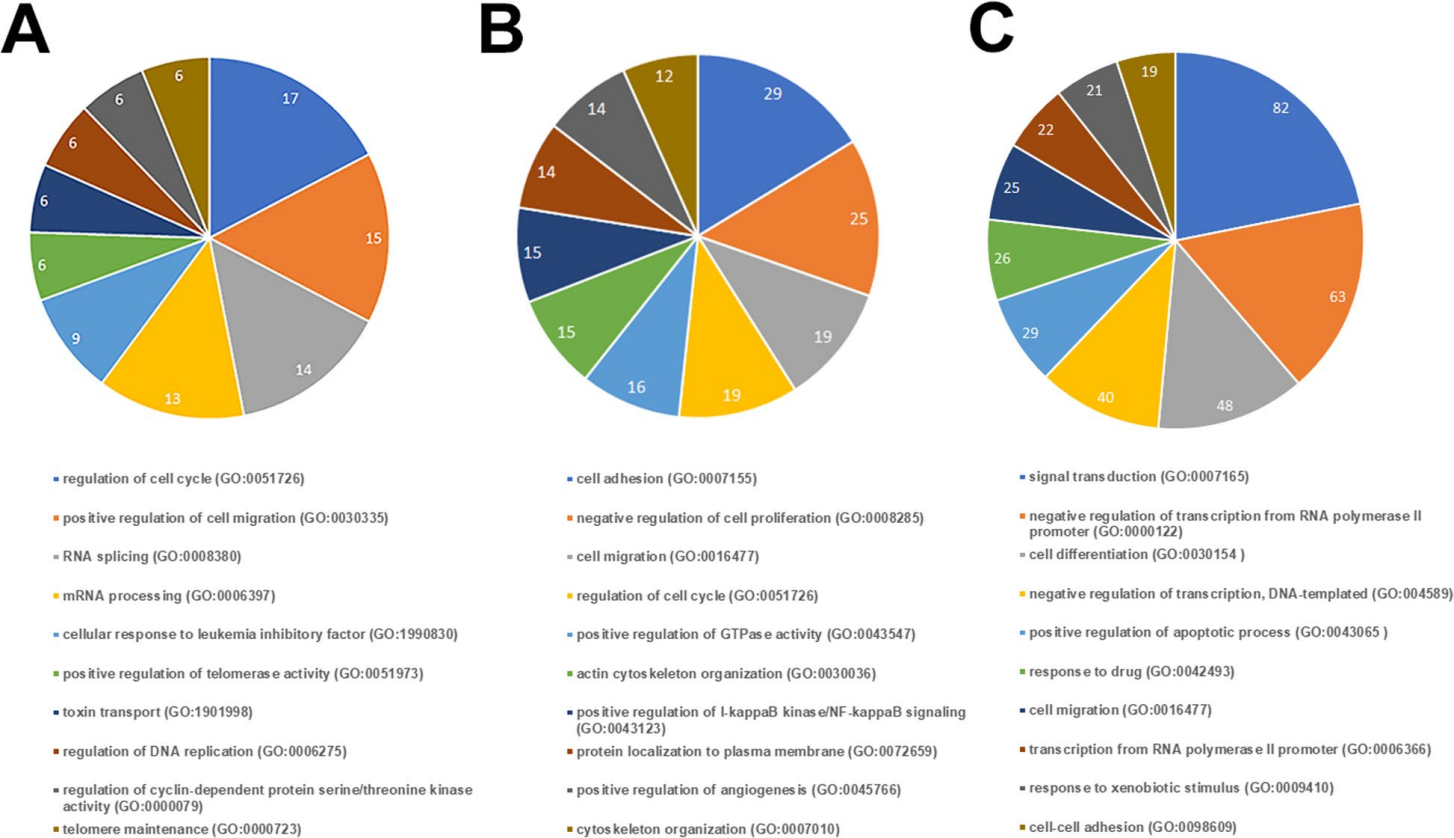
First ten pathways in induced tumors. **A** GO term enrichment analysis of biological processes that were significantly over-represented when considering differentially expressed genes in shRNA treated-tumors. **B** GO term enrichment analysis of biological processes that were significantly over-represented when considering differentially expressed genes in bevacizumab-treated tumors. **C** GO term enrichment analysis of biological processes that were significantly over-represented when considering differentially expressed genes in shRNA-plus bevacizumab-treated tumors. The remaining pathways are listed in Supplementary Table S8

PRJNA635121 bioproject (https://www.ncbi.nlm.nih.gov/bioproject/635121) contains the expression profiles of CRC patients before and after bevacizumab treatment. Interestingly, *SMC1A* is overexpressed in untreated CRC samples (data not shown) and following bevacizumab treatment there were sixteen dysregulated genes in common with our bevacizumab-treated tumors (Supplementary Table S6). Dysregulated genes were analyzed by the DAVID tool for classification by molecular function and biological process. In shRNA-treated tumors, most of the identified pathways are related to mRNA processing (GO:0006397, GO:0008380; GO:1,903,241), cell cycle regulation (GO:0051726), telomere metabolism (GO:0000723, GO:0051973), and positive regulation of cell migration (GO:0030335) (Fig. 4A). Treatment with bevacizumab, instead, involved cell proliferation and cell cycle (GO:0008285, GO:0051726, and GO:0030308), cytoskeleton organization (GO:0030036 and GO:0007010) and, as expected, regulation of angiogenesis (GO:0045766) (Fig. 4B). The combo treatment increased the number of dysregulated genes by nearly one and half times, 1014 (555 down and 459 up, Supplementary Table S7, Supplementary Figure S2). Dysregulated genes belong to many biological processes, in particular signal transduction (GO:0007165), transcription regulation (GO:0000122, GO:004589, GO:0006366, GO:0006354 and GO:0006351), regulation of apoptosis (GO:0043065), cell growth and differentiation (GO:0030154, GO:0030308 and GO:0030307), and regulation of angiogenesis (GO:0045766) (Fig. 4C). The complete list of dysregulated pathways is reported in Supplementary Table S8. Furthermore, 52 dysregulated genes were shared in common among the three groups (Fig. 3D). Fifty-one out of 52 genes (98%) maintained the same trend though to a different extent (Supplementary Figure S4). RNA-seq data were validated for ten genes by qPCR experiments (Supplementary Figure S5). These genes were chosen because most of them are involved in CRC development and their differential expression could explain the increased lifespan of mice.

It is well-known that chromosome aneuploidy leads to change in gene expression. Therefore, the chromosome status of 30 samples (7 *SMC1A*-Ov, 8 deriving from shRNA treatment, 8 from bevacizumab treatment and 7 from combo treatment) was analyzed by array Comparative Genomic Hybridization (CGH) as we previously described [44]. We found that gene dysregulation did not depend on chromosome imbalance. Indeed, CGH array revealed no chromosome gain or loss in all analyzed tumors (data not shown).

This finding indicates that the increased survival of the mice induced by shRNA and bevacizumab is associated with the alteration of specific biological pathways, predominantly involved in cell cycle, mRNA processing and gene transcription regulation, without affecting specific chromosome balance.

### Abnormal mitotic figures in vivo and in vitro

**Fig. 5:**
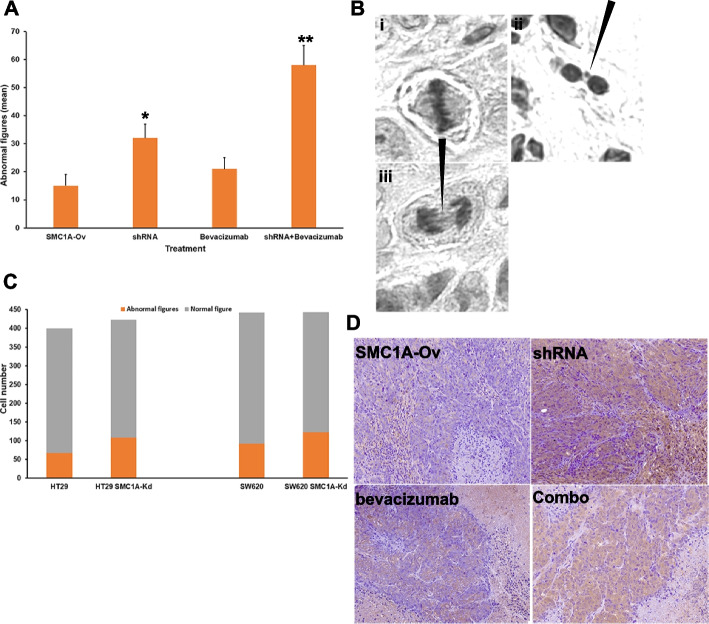
Abnormal mitotic figures and apoptosis in vitro and in vivo. **A** Tumors induced by shRNA, bevacizumab and combined treatment show high frequency of atypical mitotic figures (mean ± SD). **B** Morphological features of atypical mitosis. A normal metaphase plate (i), a spontaneous micronucleus (ii, black arrow) and lagging chromosomes (iii, black arrow). These images are representative of combo treatment. **C** Mitotic abnormal figures are a characteristic of CRC cell lines. Both HT29 and SW620 show high levels of atypical figures. **D** Apoptotic cells detected by using an anti-Caspase 3 antibody in *SMC1A*-Ov, shRNA-, bevacizumab- and combo-treated cells. **p < 0.01, *p < 0.05

Atypical mitoses are characterized by abnormal sister chromatid separation and abnormalities in the mitotic spindle symmetry and are thought to reflect genetic alterations that underlie the malignant phenotype. In view of the paramount importance of abnormal mitotic figures, we analyzed their frequencies in our tumors. The median of atypical mitoses ranged from 15 to 58. Notably, the distribution of typical and atypical mitoses was not uniform between tumors. In fact, tumors derived from *SMC1A*-Ov showed low levels of atypical mitoses while tumors treated with the combo showed a high proportion of atypical mitoses compared to the overall number of mitoses (Fig. 5A& B). These data along with the ones showing that abnormal figures after *SMC1A* knockdown reached a peak after 4 days in cell culture (Fig. 1D), prompted us to investigate whether this observation is peculiar to HCT116 cells, used in this study, or is a more general phenomenon. To this aim, two additional colon cancer cell lines, HT29 and SW620, were transfected with the *SMC1A*-specific shRNA. Data showed that both cell lines displayed high numbers of atypical figures (Fig. 5C), suggesting that abnormal mitosis is a feature of CRC cells after *SMC1A* downregulation.

Finally, apoptosis frequency was analyzed in tumors using a Caspase 3 antibody. We found that tumors derived from treatment with *SMC1A*-specific shRNA and bevacizumab displayed a significant increase in apoptosis (*p* = 0.037 and *p* = 0.023, respectively) when compared with *SMC1A*-Ov tumors. This amount of apoptosis was not further increased by the combo treatment, bevacizumab plus shRNA against *SMC1A* (Fig. 5D).

These results indicate that both *SMC1A* silencing and combo treatment lead to significant levels of atypical mitotic figures which in turn could trigger the apoptotic process.

## Discussion

Recent evidence suggests that *SMC1A* gene, coding for a member of the cohesin complex, is implicated in CRC. Indeed, *SMC1A* mutations have been identified in CRC [26, 27] whereas overexpression of the protein has been found in advanced diseases and is associated with a poor prognosis [28, 29], suggesting that inhibition of *SMC1A* may serve as a promising therapeutic strategy for human CRC.

In the present study, shRNA was employed to silence the expression of *SMC1A* in HCT116 CRC cells. Downregulation of *SMC1A* induced decreased cell proliferation when compared to mock- and *SMC1A*-overexpressing cells. Our results are concordant with the outcome of *SMC1A* knocking down in both glioma and lung adenocarcinoma cells, in which cell proliferation was suppressed through G1/S or G2/M phase cell cycle arrest [45, 46]. A notable result of these studies is that the reduction of about 90% of total *SMC1A* expression causes the impairment of cell proliferation. On the contrary, *SMC1A* overexpression does not affect cell cycle progression. These results are consistent with previous estimates that about 15% of cohesin is required to maintain proper cell cycle progression and proliferation [47]. *SMC1A* knockdown also induces abnormal mitotic figures with multipolar spindles or altered DNA distribution. Interestingly, this observation is not limited to HCT116 cells, but atypical mitoses have been found in two additional CRC cell lines, HT29 and SW620, suggesting that this is a general phenomenon of CRC cells. In addition, this finding suggests that atypical mitoses are p*53*-independent since HCT116 and SW620 cell lines harbor a wild-type *p53* while HT29 carries a mutated one. Since SMC1A associates with mitotic microtubules at the spindle pole [48], this data indicates that imbalances in the concentration of cohesin subunits in the mitotic spindle formation pathways interfere with the assembly of normal bipolar spindles.

**Fig. 6:**
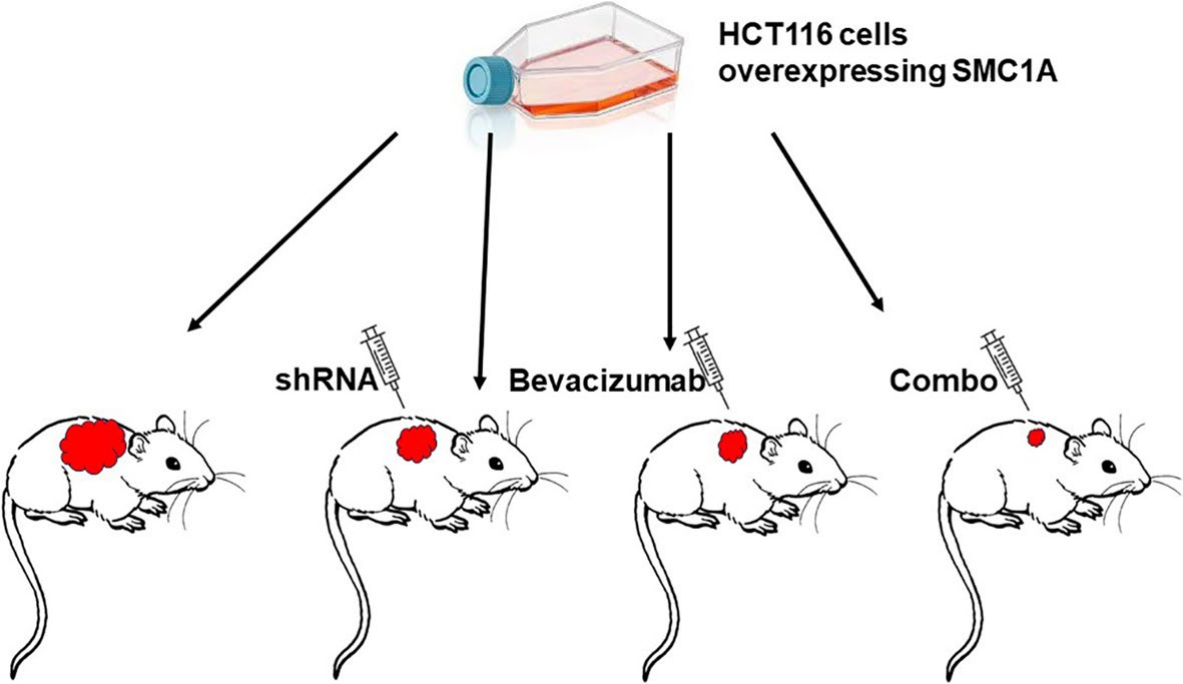
*SMC1A* and colorectal cancer. The overexpression of *SMC1A* reduced the latency period of cancer formation in a subcutaneous murine xenograft model and the volume of tumors were significantly increased in presence of upregulated *SMC1A*. The subsequent shRNA-mediated *SMC1A* silencing effectively downregulated cancer progression and this effect was enhanced following the combined treatment, shRNA against *SMC1A* plus bevacizumab

Next, we investigated the effects in vivo of *SMC1A* silencing on the development of tumor xenografts in immunodeficient mice (Fig. 6). The volume of tumors was significantly decreased upon silencing of the *SMC1A* gene while its upregulation had a positive impact on cancer progression. In addition, median overall survival was significantly higher following *SMC1A* silencing compared with the control group. This result indicates that shRNA-mediated *SMC1A* silencing effectively downregulates CRC progression in an in vivo model. It is worth noting that progression-free survival was higher for bevacizumab and combo (bevacizumab plus *SMC1A*-specific shRNA) treated mice. Bevacizumab, a monoclonal antibody against vascular endothelial growth factor, was approved for the treatment of CRC by the U.S. FDA in 2004. In addition, it has been shown to significantly improve the survival of CRC patients in combination with 5-fluorouracil-based chemotherapy [36, 49, 50]. Overall survival is a fundamental endpoint in clinical trials, since it represents the ultimate goal of available treatments and strategies. The present findings show that *SMC1A* knockdown alone or in combination with bevacizumab impairs cancer growth, translating into a consistent improvement in animals’ overall survival.

Aneuploidy, abnormality in the number of chromosomes in cells, is a very frequent feature in most human cancers. High levels of aneuploidy are associated with several parameters of aggressiveness in cancers, including resistance to therapy, metastatic spread and poor prognosis [51]. Notably, we found that tumor reduction and mouse survival induced by *SMC1A* silencing and bevacizumab are associated with high levels of abnormal mitotic figures and spontaneous micronuclei, which are markers of chromosome aneuploidy. The finding that array CGH detected no aneuploidy is not inconsistent. In fact, the detection threshold limit is around 8% as previously shown [52].

However, while the aneuploidy observed in tumors induced by the treatments with shRNA against *SMC1A* and bevacizumab alone leads to activation of the apoptotic process, the combined treatment exhibited a level of apoptosis comparable to control tumors suggesting that either cells have reached the maximum level of apoptosis or that the combo treatment triggers other processes such as necrosis. In this context, it is likely that the effects of aneuploidy may be related to the tumor microenvironment, including the immune clearance of aneuploid cells, or to timing, severity, and duration of aneuploidy. This notion is further supported by the observation that trisomic hematopoietic stem cells (HSCs) show decreased fitness compared with euploid controls when transplanted into irradiated mice. Again, aneuploid cells were depleted from the peripheral blood over time in chromosomally unstable BubR1^H/H^ HSCs [53]. Our observations made both in vitro and in vivo suggest that aneuploidy is detrimental to cell proliferation. This fitness defect arises because of changes in the copy number of genes located on the aneuploid chromosomes which in turn alters the production of hundreds of proteins.

*SMC1A* silencing and bevacizumab (alone or in combination) resulted in significant changes in gene expression profiles. We found 697, 762 and 1014 dysregulated genes following shRNA, bevacizumab and combo treatments, respectively. These differentially expressed genes were virtually implicated in many metabolic pathways, including cell cycle, gene transcription regulation and mRNA processing. The availability of RNA-seq data obtained from CRC patients treated with bevacizumab (see PRJNA635121 bioproject) allowed us to identify sixteen dysregulated genes that are in common with our bevacizumab-treated tumors. Of note, eight of them, namely *CLIP4*, *GSTM2*, *HTR1D*, *LTB*, *PDLIM2*, *RASSF2*, *SAA1* and *SAA2 *have been implicated in CRC development [54–62]. All treatments share a subset of 52 dysregulated genes. Thus, the mechanisms of *SMC1A* knockdown (alone or in combination with bevacizumab) restricting CRC cell growth may occur, in part, through the dysregulation of specific pathways.

## Conclusion

Collectively, our data suggest that in the absence of *SMC1A*, cell proliferation and tumor development were efficiently suppressed. Therefore, shRNA-mediated *SMC1A* silencing could be a valuable therapeutic approach for CRC treatment. In addition to targeting the cell division machinery, it may also be possible to expose the consequence of cell division errors by exploiting vulnerabilities associated with the aneuploid state itself. Aneuploid cells are more sensitive than euploid cells to compounds that exacerbate metabolic stress. In this context, the combined treatment with bevacizumab acts synergistically to suppress the growth of xenograft tumors. Together, this study offers a proof of principle that cohesin complex can be exploited therapeutically and open the door to the possibility of generating broad-spectrum anti-cancer drugs that aim to exacerbate stresses inherent to chromosomally instable tumors, such as CRC.

### Supplementary Information

**Additional file 1: Supplementary Fig. 1.** A Effects of both *SMC1A* upregulation and downregulation *in vitro*. A Transfections with vectors overexpressing or silencing *SMC1A* lead to the overexpression of SMC1A protein or its downregulation when compared to mock cells 24 h after the transfection. Tubulin antibody was used as loading control. B* SMC1A* inhibition causes a significant frequency of mitotic abnormal figures when compared to untreated and *SMC1A*-Ov cells. *p < 0.05.

**Additional file 2: Supplementary Fig. 2.** RNA-seq analysis. Number of dysregulated genes in shRNA, bevacizumab and combo tumors.

**Additional file 3: Supplementary Fig. 3.** RNA-seq analysis. A Volcano plot of shRNA tumors. B Volcano plot of bevacizumab-treated tumors. C Volcano plot of shRNA- and bevacizumab-treated tumors.

**Additional file 4: Supplementary Fig. 4.** RNA-seq analysis. Heatmap of fifty-two dysregulated genes.

**Additional file 5: Supplementary Fig. 5.** RNA-seq analysis. Gene expression profile data was validated by RT-qPCR. *p < 0.05.

**Additional file 6: Table S1.** Primers sequences used for validating RNA-seq data by RT-qPCR.

**Additional file 7: Table S2.** Relative plating efficiency (RPE) of untreated, *SMC1A*-Ov and *SMC1A*-Kd cells.

**Additional file 8: Table S3.** Pairwise comparison between *SMC1A*-Ov, shRNA, bevacizumab and combo treatments.

**Additional file 9: Table S4.** Dysregulated genes (down- and upregulated) following shRNA treatment.

**Additional file 10: Table S5.** Dysregulated genes (down- and upregulated) following bevacizumab treatment.

**Additional file 11: Table S6.** Common dysregulated genes between PRJNA635121 bioproject and our bevacizumab-treated tumors.

**Additional file 12: Table S7.** Dysregulated genes (down- and upregulated) following combo treatment.

**Additional file 13: Table S8.** Dysregulated pathways following shRNA, bevacizumab and combo treatments.

## Data Availability

The relevant data supporting the findings of this study are available in this article and its supplementary information files. All NGS raw files have been deposited into NCBI Sequence Read Archive under accession number PRJNA1016101.

## Abbreviations

aCGH: Array Comparative Genomic Hybridization
CIN: Chromosomal instability
CRC: Colorectal cancer
DAVID: Database for Annotation, Visualization, and Integrated Discovery
DMEM: Dulbecco’s minimal essential medium
FDA: Food and Drug Administration
LOH: Loss of heterozygosity
MSI: Microsatellite instability
PCA: Principal component analysis
qPCR: Quantitative real-time PCR
RNA-seq: RNA-sequencing
RPE: Relative plating efficiency
SD: Standard deviation
VEGF-A: Vascular Endothelial Growth Factor A

## References

[1] Sung H, Ferlay J, Siegel RL, Laversanne M, Soerjomataram I, Jemal A, Bray F (2021). Global Cancer Statistics 2020: GLOBOCAN estimates of incidence and mortality worldwide for 36 cancers in 185 countries. CA Cancer J Clin.

[2] Siegel RL, Miller KD, Fuchs HE, Jemal A (2021). Cancer statistics, 2021. CA Cancer J Clin.

[3] Marmol I, Sanchez-de-Diego C, PradillaDieste A, Cerrada E, Rodriguez Yoldi MJ (2017). Colorectal carcinoma: a general overview and future perspectives in colorectal cancer. Int J Mol Sci.

[4] Pino MS, Chung DC (2010). The chromosomal instability pathway in colon cancer. Gastroenterology.

[5] Horsfield JA (2023). Full circle: a brief history of cohesin and the regulation of gene expression. FEBS J.

[6] Di Nardo M, Pallotta MM, Musio A (2022). The multifaceted roles of cohesin in cancer. J Exp Clin Cancer Res.

[7] Perea-Resa C, Wattendorf L, Marzouk S, Blower MD (2021). Cohesin: behind dynamic genome topology and gene expression reprogramming. Trends Cell Biol.

[8] Zhu HE, Li T, Shi S, Chen DX, Chen W, Chen H (2021). ESCO2 promotes lung adenocarcinoma progression by regulating hnRNPA1 acetylation. J Exp Clin Cancer Res.

[9] Koedoot E, van Steijn E, Vermeer M, Gonzalez-Prieto R, Vertegaal ACO, Martens JWM, Le Devedec SE, van de Water B (2021). Splicing factors control triple-negative breast cancer cell mitosis through SUN2 interaction and sororin intron retention. J Exp Clin Cancer Res.

[10] Oishi Y, Nagasaki K, Miyata S, Matsuura M, Nishimura SI, Akiyama F, Iwai T, Miki Y (2007). Functional pathway characterized by gene expression analysis of supraclavicular lymph node metastasis-positive breast cancer. J Hum Genet.

[11] Balbas-Martinez C, Sagrera A, Carrillo-de-Santa-Pau E, Earl J, Marquez M, Vazquez M, Lapi E, Castro-Giner F, Beltran S, Bayes M (2013). Recurrent inactivation of STAG2 in bladder cancer is not associated with aneuploidy. Nat Genet.

[12] Solomon DA, Kim JS, Bondaruk J, Shariat SF, Wang ZF, Elkahloun AG, Ozawa T, Gerard J, Zhuang D, Zhang S (2013). Frequent truncating mutations of STAG2 in bladder cancer. Nat Genet.

[13] Guo G, Sun X, Chen C, Wu S, Huang P, Li Z, Dean M, Huang Y, Jia W, Zhou Q (2013). Whole-genome and whole-exome sequencing of bladder cancer identifies frequent alterations in genes involved in sister chromatid cohesion and segregation. Nat Genet.

[14] Taylor CF, Platt FM, Hurst CD, Thygesen HH, Knowles MA (2014). Frequent inactivating mutations of STAG2 in bladder cancer are associated with low tumour grade and stage and inversely related to chromosomal copy number changes. Hum Mol Genet.

[15] Cancer Genome Atlas Research N (2014). Comprehensive molecular characterization of urothelial bladder carcinoma. Nature.

[16] Brennan CW, Verhaak RG, McKenna A, Campos B, Noushmehr H, Salama SR, Zheng S, Chakravarty D, Sanborn JZ, Berman SH (2013). The somatic genomic landscape of glioblastoma. Cell.

[17] Bailey ML, O'Neil NJ, van Pel DM, Solomon DA, Waldman T, Hieter P (2014). Glioblastoma cells containing mutations in the cohesin component STAG2 are sensitive to PARP inhibition. Mol Cancer Ther.

[18] Crompton BD, Stewart C, Taylor-Weiner A, Alexe G, Kurek KC, Calicchio ML, Kiezun A, Carter SL, Shukla SA, Mehta SS (2014). The genomic landscape of pediatric Ewing sarcoma. Cancer Discov.

[19] Brohl AS, Solomon DA, Chang W, Wang J, Song Y, Sindiri S, Patidar R, Hurd L, Chen L, Shern JF (2014). The genomic landscape of the Ewing Sarcoma family of tumors reveals recurrent STAG2 mutation. Plos Genet.

[20] Tirode F, Surdez D, Ma X, Parker M, Le Deley MC, Bahrami A, Zhang Z, Lapouble E, Grossetete-Lalami S, Rusch M (2014). Genomic landscape of Ewing sarcoma defines an aggressive subtype with co-association of STAG2 and TP53 mutations. Cancer Discov.

[21] Ryu B, Kim DS, Deluca AM, Alani RM (2007). Comprehensive expression profiling of tumor cell lines identifies molecular signatures of melanoma progression. Plos One.

[22] Kon A, Shih LY, Minamino M, Sanada M, Shiraishi Y, Nagata Y, Yoshida K, Okuno Y, Bando M, Nakato R (2013). Recurrent mutations in multiple components of the cohesin complex in myeloid neoplasms. Nat Genet.

[23] Thota S, Viny AD, Makishima H, Spitzer B, Radivoyevitch T, Przychodzen B, Sekeres MA, Levine RL, Maciejewski JP (2014). Genetic alterations of the cohesin complex genes in myeloid malignancies. Blood.

[24] Thol F, Bollin R, Gehlhaar M, Walter C, Dugas M, Suchanek KJ, Kirchner A, Huang L, Chaturvedi A, Wichmann M (2014). Mutations in the cohesin complex in acute myeloid leukemia: clinical and prognostic implications. Blood.

[25] Papaemmanuil E, Gerstung M, Bullinger L, Gaidzik VI, Paschka P, Roberts ND, Potter NE, Heuser M, Thol F, Bolli N (2016). Genomic classification and prognosis in acute Myeloid Leukemia. N Engl J Med.

[26] Barber TD, McManus K, Yuen KW, Reis M, Parmigiani G, Shen D, Barrett I, Nouhi Y, Spencer F, Markowitz S (2008). Chromatid cohesion defects may underlie chromosome instability in human colorectal cancers. Proc Natl Acad Sci U S A.

[27] Cucco F, Servadio A, Gatti V, Bianchi P, Mannini L, Prodosmo A, De Vitis E, Basso G, Friuli A, Laghi L (2014). Mutant cohesin drives chromosomal instability in early colorectal adenomas. Hum Mol Genet.

[28] Wang J, Yu S, Cui L, Wang W, Li J, Wang K, Lao X (2015). Role of SMC1A overexpression as a predictor of poor prognosis in late stage colorectal cancer. BMC Cancer.

[29] Sarogni P, Palumbo O, Servadio A, Astigiano S, D'Alessio B, Gatti V, Cukrov D, Baldari S, Pallotta MM, Aretini P (2019). Overexpression of the cohesin-core subunit SMC1A contributes to colorectal cancer development. J Exp Clin Cancer Res.

[30] Kim ST, Xu B, Kastan MB (2002). Involvement of the cohesin protein, Smc1, in Atm-dependent and independent responses to DNA damage. Genes Dev.

[31] Yazdi PT, Wang Y, Zhao S, Patel N, Lee EY, Qin J (2002). SMC1 is a downstream effector in the ATM/NBS1 branch of the human S-phase checkpoint. Genes Dev.

[32] Musio A, Montagna C, Mariani T, Tilenni M, Focarelli ML, Brait L, Indino E, Benedetti PA, Chessa L, Albertini A (2005). SMC1 involvement in fragile site expression. Hum Mol Genet.

[33] Kitagawa R, Bakkenist CJ, McKinnon PJ, Kastan MB (2004). Phosphorylation of SMC1 is a critical downstream event in the ATM-NBS1-BRCA1 pathway. Genes Dev.

[34] Musio A (2020). The multiple facets of the SMC1A gene. Gene.

[35] Maiorano BA, Parisi A, Maiello E, Ciardiello D (2022). The interplay between anti-angiogenics and immunotherapy in colorectal cancer. Life (Basel).

[36] Hurwitz H, Fehrenbacher L, Novotny W, Cartwright T, Hainsworth J, Heim W, Berlin J, Baron A, Griffing S, Holmgren E (2004). Bevacizumab plus irinotecan, fluorouracil, and leucovorin for metastatic colorectal cancer. N Engl J Med.

[37] Musio A, Montagna C, Zambroni D, Indino E, Barbieri O, Citti L, Villa A, Ried T, Vezzoni P (2003). Inhibition of BUB1 results in genomic instability and anchorage-independent growth of normal human fibroblasts. Cancer Res.

[38] Mannini L, Cucco F, Quarantotti V, Amato C, Tinti M, Tana L, Frattini A, Delia D, Krantz ID, Jessberger R (2015). SMC1B is present in mammalian somatic cells and interacts with mitotic cohesin proteins. Sci Rep.

[39] Mannini L, Lamaze FC, Cucco F, Amato C, Quarantotti V, Rizzo IM, Krantz ID, Bilodeau S, Musio A (2015). Mutant cohesin affects RNA polymerase II regulation in Cornelia de lange syndrome. Sci Rep.

[40] Maiato H, Logarinho E (2014). Mitotic spindle multipolarity without centrosome amplification. Nat Cell Biol.

[41] Kramer A, Maier B, Bartek J (2011). Centrosome clustering and chromosomal (in)stability: a matter of life and death. Mol Oncol.

[42] Mori S, Chang JT, Andrechek ER, Matsumura N, Baba T, Yao G, Kim JW, Gatza M, Murphy S, Nevins JR (2009). Anchorage-independent cell growth signature identifies tumors with metastatic potential. Oncogene.

[43] Liu Y, Fang X, Wang Q, Xiao D, Zhou T, Kang K, Peng Z, Ren F, Zhou J (2023). SMC1A facilitates gastric cancer cell proliferation, migration, and invasion via promoting SNAIL activated EMT. BMC Gastroenterol.

[44] Barone S, Sarogni P, Valli R, Pallotta MM, Silvia G, Frattini A, Khan AW, Rapalini E, Parri C, Musio A (2020). Chromosome missegregation in single human oocytes is related to the age and gene expression profile. Int J Mol Sci.

[45] Zhang YF, Jiang R, Li JD, Zhang XY, Zhao P, He M, Zhang HZ, Sun LP, Shi DL, Zhang GX (2013). SMC1A knockdown induces growth suppression of human lung adenocarcinoma cells through G1/S cell cycle phase arrest and apoptosis pathways in vitro. Oncol Lett.

[46] Ma Z, Lin M, Li K, Fu Y, Liu X, Yang D, Zhao Y, Zheng J, Sun B (2013). Knocking down SMC1A inhibits growth and leads to G2/M arrest in human glioma cells. Int J Clin Exp Pathol.

[47] Laugsch M, Seebach J, Schnittler H, Jessberger R (2013). Imbalance of SMC1 and SMC3 cohesins causes specific and distinct effects. Plos One.

[48] Wong RW, Blobel G (2008). Cohesin subunit SMC1 associates with mitotic microtubules at the spindle pole. Proc Natl Acad Sci U S A.

[49] Cremolini C, Loupakis F, Antoniotti C, Lupi C, Sensi E, Lonardi S, Mezi S, Tomasello G, Ronzoni M, Zaniboni A (2015). FOLFOXIRI plus bevacizumab versus FOLFIRI plus bevacizumab as first-line treatment of patients with metastatic colorectal cancer: updated overall survival and molecular subgroup analyses of the open-label, phase 3 TRIBE study. Lancet Oncol.

[50] Loupakis F, Cremolini C, Masi G, Lonardi S, Zagonel V, Salvatore L, Cortesi E, Tomasello G, Ronzoni M, Spadi R (2014). Initial therapy with FOLFOXIRI and bevacizumab for metastatic colorectal cancer. N Engl J Med.

[51] Levine MS, Holland AJ (2018). The impact of mitotic errors on cell proliferation and tumorigenesis. Genes Dev.

[52] Valli R, Marletta C, Pressato B, Montalbano G, Lo Curto F, Pasquali F, Maserati E (2011). Comparative genomic hybridization on microarray (a-CGH) in constitutional and acquired mosaicism may detect as low as 8% abnormal cells. Mol Cytogenet.

[53] Pfau SJ, Silberman RE, Knouse KA, Amon A (2016). Aneuploidy impairs hematopoietic stem cell fitness and is selected against in regenerating tissues in vivo. Genes Dev.

[54] Wen L, Han Z, Du Y (2021). Identification of gene biomarkers and immune cell infiltration characteristics in rectal cancer. J Gastrointest Oncol.

[55] Zeng C, Chen Y (2019). HTR1D, TIMP1, SERPINE1, MMP3 and CNR2 affect the survival of patients with colon adenocarcinoma. Oncol Lett.

[56] Wu B, Yang J, Qin Z, Yang H, Shao J, Shang Y (2022). Prognosis prediction of stage IV colorectal cancer patients by mRNA transcriptional profile. Cancer Med.

[57] Zeng Y, Lin D, Gao M, Du G, Cai Y (2022). Systematic evaluation of the prognostic and immunological role of PDLIM2 across 33 cancer types. Sci Rep.

[58] Wu Y, Wan X, Jia G, Xu Z, Tao Y, Song Z, Du T (2020). Aberrantly Methylated and expressed genes as prognostic epigenetic biomarkers for colon cancer. DNA Cell Biol.

[59] Riffet M, Eid Y, Faisant M, Fohlen A, Menahem B, Alves A, Dubois F, Levallet G, Bazille C (2021). Deciphering promoter Hypermethylation of genes encoding for RASSF/Hippo pathway reveals the poor prognostic factor of RASSF2 gene silencing in colon cancers. Cancers (Basel).

[60] Carter JV, O'Brien SJ, Burton JF, Oxford BG, Stephen V, Hallion J, Bishop C, Galbraith NJ, Eichenberger MR, Sarojini H (2019). The microRNA-200 family acts as an oncogene in colorectal cancer by inhibiting the tumor suppressor RASSF2. Oncol Lett.

[61] Sun R, Yang Y, Lu W, Yang Y, Li Y, Liu Z, Diao D, Wang Y, Chang S, Lu M (2023). Single-cell transcriptomic analysis of normal and pathological tissues from the same patient uncovers colon cancer progression. Cell Biosci.

[62] Zhang W, Shi Y, Niu S, Li L, Lin L, Gao X, Cai W, Chen Y, Zhong Y, Tang D (2022). Integrated computer analysis and a self-built Chinese cohort study identified GSTM2 as one survival-relevant gene in human colon cancer potentially regulating immune microenvironment. Front Oncol.

