# Supplementary figures and images for: The synergism of SMC1A cohesin gene silencing and bevacizumab against colorectal cancer
Source: J Exp Clin Cancer Res. 2024 Feb 16;43:49. doi: 10.1186/s13046-024-02976-2 (PMC10870497; doi:10.1186/s13046-024-02976-2)

A

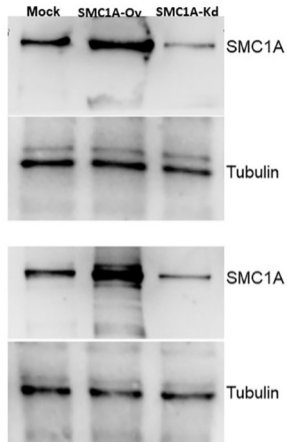

B

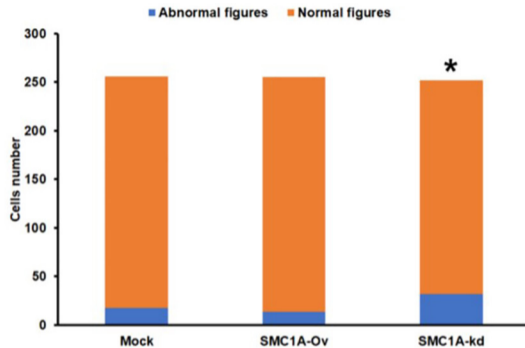

Fig. S1

Supplement: Supplementary file 1 — Additional file 1: Supplementary Fig. 1. A Effects of both SMC1A upregulation and downregulation in vitro. A Transfections with vectors overexpressing or silencing SMC1A lead to the overexpression of SMC1A protein or its downregulation when compared to mock cells 24 h after the transfection. Tubulin antibody was used as loading control. B SMC1A inhibition causes a significant frequency of mitotic abnormal figures when compared to untreated and SMC1A-Ov cells. *p < 0.05. [file 13046_2024_2976_MOESM1_ESM.pdf]

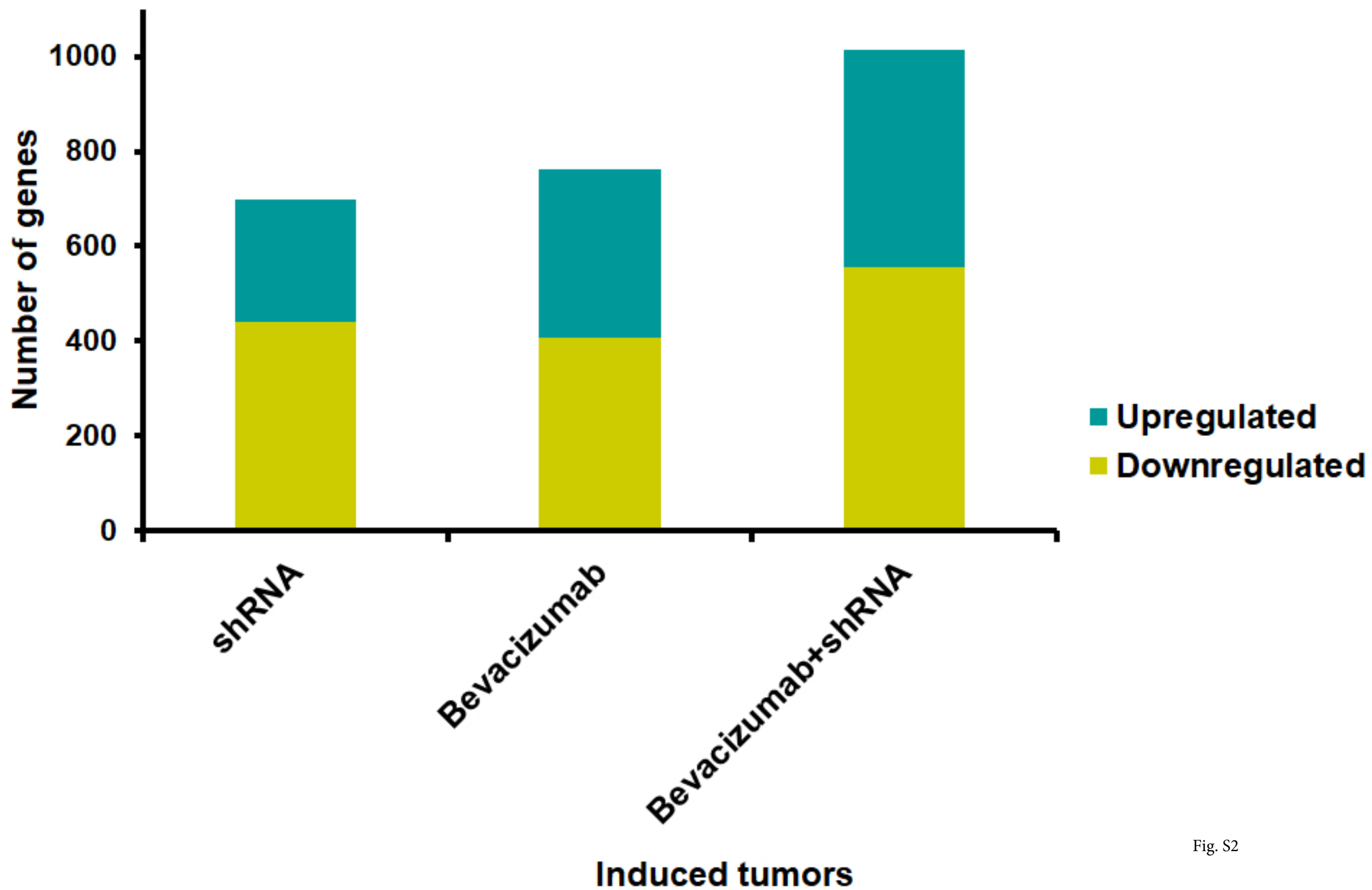

Fig. S2

Supplement: Supplementary file 2 — Additional file 2: Supplementary Fig. 2. RNA-seq analysis. Number of dysregulated genes in shRNA, bevacizumab and combo tumors. [file 13046_2024_2976_MOESM2_ESM.pdf]

**A**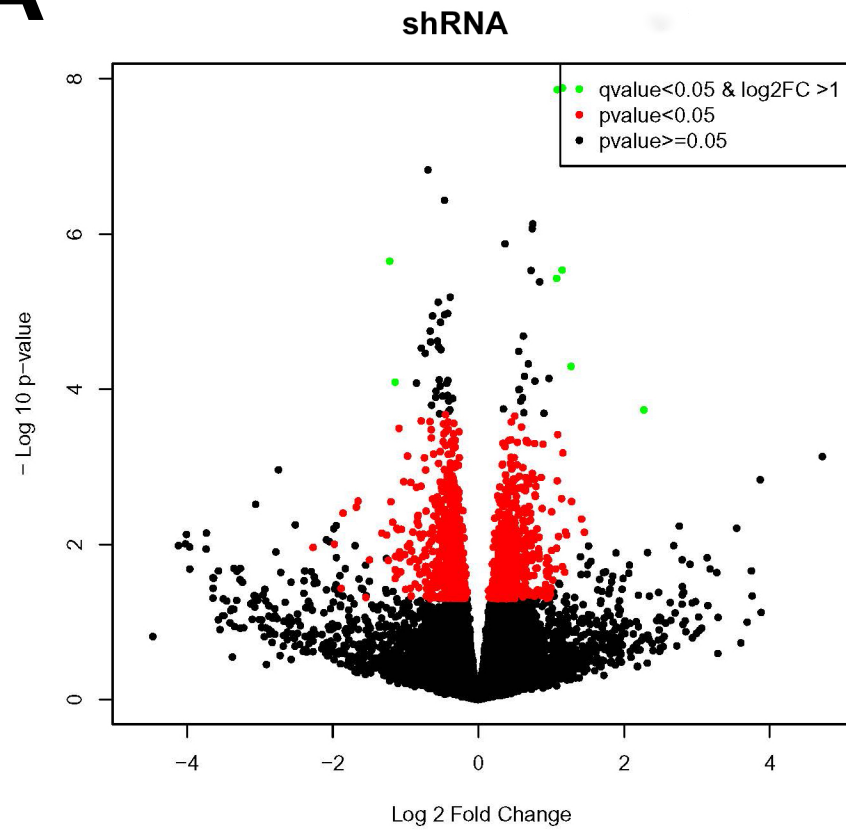**B**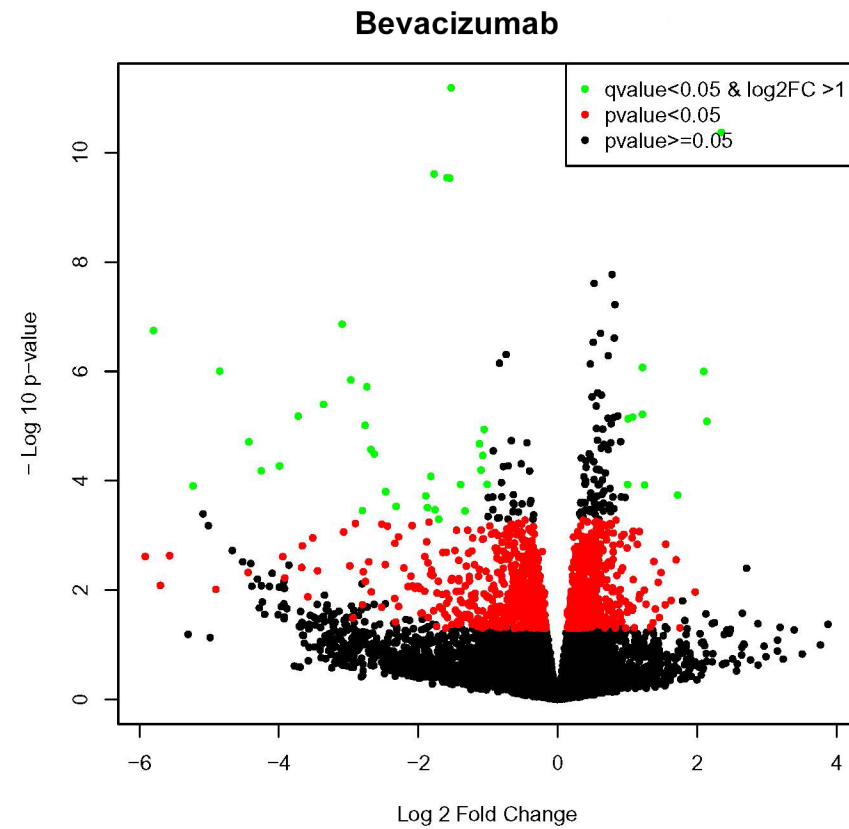**C**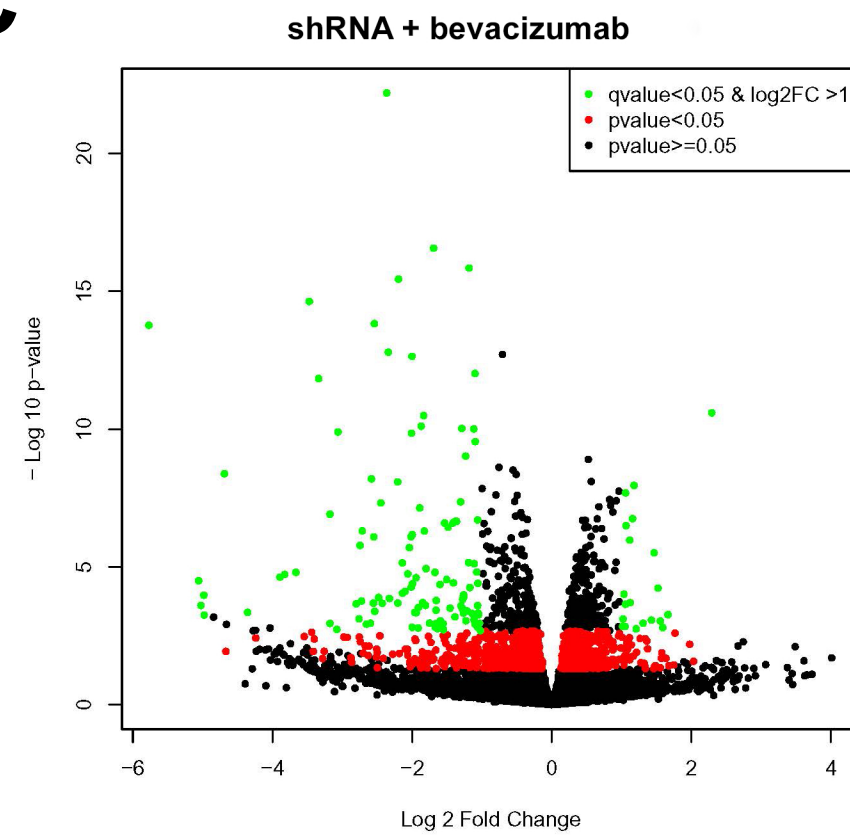

Fig. S3

Supplement: Supplementary file 3 — Additional file 3: Supplementary Fig. 3. RNA-seq analysis. A Volcano plot of shRNA tumors. B Volcano plot of bevacizumab-treated tumors. C Volcano plot of shRNA- and bevacizumab-treated tumors. [file 13046_2024_2976_MOESM3_ESM.pdf]

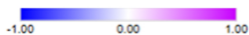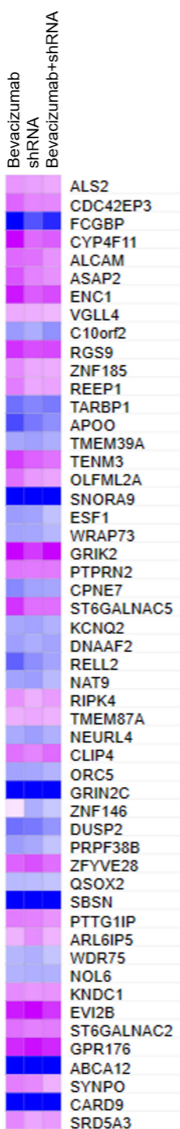

Fig. S4

Supplement: Supplementary file 4 — Additional file 4: Supplementary Fig. 4. RNA-seq analysis. Heatmap of fifty-two dysregulated genes. [file 13046_2024_2976_MOESM4_ESM.pdf]

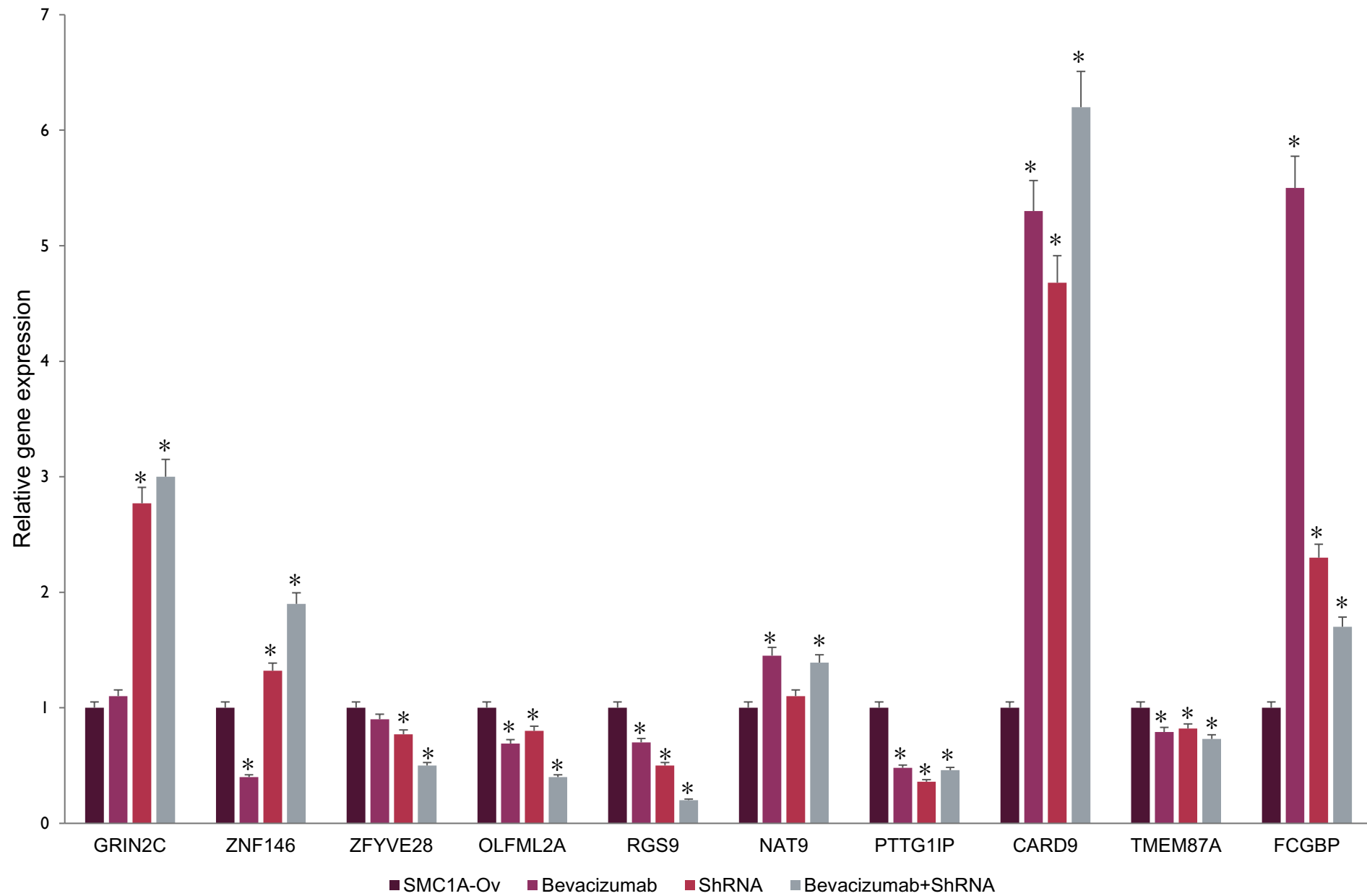

Fig. S5

Supplement: Supplementary file 5 — Additional file 5: Supplementary Fig. 5. RNA-seq analysis. Gene expression profile data was validated by RT-qPCR. *p < 0.05. [file 13046_2024_2976_MOESM5_ESM.pdf]
