# Supplementary material for: The synergism of SMC1A cohesin gene silencing and bevacizumab against colorectal cancer
Source: J Exp Clin Cancer Res. 2024 Feb 16;43:49. doi: 10.1186/s13046-024-02976-2 (PMC10870497; doi:10.1186/s13046-024-02976-2)
Supplement: Supplementary file 6 — Additional file 6: Table S1. Primers sequences used for validating RNA-seq data by RT-qPCR. [file 13046_2024_2976_MOESM6_ESM.pdf]

Table S1. Primers sequences used for validating RNA-seq data by RT-qPCR

Gene	Forward primer sequence (5'-3')	Reverse primer sequence (5'-3')
GRIN2C	TCTTGAACTACAAGGCCGGG	GCGAGGTCAATCTGCCTCTT
ZNF146	GGCGCCAAAGTAGGAGACTT	CATTCTTACCAGGCGTAGGCT
ZFYVE28	CAACTGCTGGCCCAGTTCTA	ACGGTCCTGAGGGATACACT
OLFML2A	CCCCTGTGAAACACCACAGT	CACCAGGCTGTTTCCGTAGT
RGS9	GAGTCGTGCACGTAGGAGAG	CTAAAGCAATGTGGCTGGGC
NAT9	GAGTGACCACGCTAGGTCTG	TGCTCCAGAAGCCACTGATG
PTTG1IP	CCAGTTACAAGCGTCTTGCC	CGCCTCAAAGTTCACCCAAC
CARD9	GAGGACCGGGAGAACACCA	CCAGATTCCTCGTTCCAGGC
TMEM87A	TTCCGATACCGTCGGGGAAA	CAGCGCTTTTCAGATACCAGG
FCGBP	CTCCACAGTGCCAGGATGG	ACCATGCCTTTACCCGCA
