# Supplementary material for: The synergism of SMC1A cohesin gene silencing and bevacizumab against colorectal cancer
Source: J Exp Clin Cancer Res. 2024 Feb 16;43:49. doi: 10.1186/s13046-024-02976-2 (PMC10870497; doi:10.1186/s13046-024-02976-2)
Supplement: Supplementary file 7 — Additional file 7: Table S2. Relative plating efficiency (RPE) of untreated, SMC1A-Ov and SMC1A-Kd cells. [file 13046_2024_2976_MOESM7_ESM.pdf]

Table S2. Relative plating efficiency (RPE) of untreated, *SMC1A*-Ov and *SMC1A*-Kd cells.

Samples	RPE (%)
Untreated	98.7
SMC1A-Ov	84.8
SMC1A-Kd	49.4
