# Supplementary material for: The synergism of SMC1A cohesin gene silencing and bevacizumab against colorectal cancer
Source: J Exp Clin Cancer Res. 2024 Feb 16;43:49. doi: 10.1186/s13046-024-02976-2 (PMC10870497; doi:10.1186/s13046-024-02976-2)
Supplement: Supplementary file 8 — Additional file 8: Table S3. Pairwise comparison between SMC1A-Ov, shRNA, bevacizumab and combo treatments. [file 13046_2024_2976_MOESM8_ESM.pdf]

Table S3. Pairwise comparison between SMC1A-ov, shRNA, bevacizumab and bevacizumab plus shRNA treatements

		SMC1A-Ov			shRNA			Bevacizumab			Bevacizumab + shRNA		
	Treatment	Chi-square	Sig.		Chi-square	Sig.		Chi-square	Sig.		Chi-square	Sig.	
Long rank (Mantel-Cox)	SMC1A-Ov				4.188	0.041		10.698	0.01		16.004	0.000	
	shRNA	4.188	0.041					1.729	0.189		3.109	0.078	
	Bevacizumab	10.698	0.01		1.729	0.189					0.192	0.661	
	Bevacizumab + shRNA	16.004	0.000		3.109	0.078		0.192	0.661				
