# Supplementary material for: The synergism of SMC1A cohesin gene silencing and bevacizumab against colorectal cancer
Source: J Exp Clin Cancer Res. 2024 Feb 16;43:49. doi: 10.1186/s13046-024-02976-2 (PMC10870497; doi:10.1186/s13046-024-02976-2)
Supplement: Supplementary file 9 — Additional file 9: Table S4. Dysregulated genes (down- and upregulated) following shRNA treatment. [file 13046_2024_2976_MOESM9_ESM.pdf]

Table S4. Dysregulated genes (down- and upregulated) following shRNA treatment.

Downregulated

**Gene**	**log2FoldChange**
HIST1H2BM	-4,11477E+14
SNORD59B	-4,0176E+14
FLJ16779	-4,00483E+14
SNORA78	-3,95946E+14
INO80B	-3,73441E+14
RNF148	-3,73007E+14
CHGB	-3,05517E+14
LEAP2	-2,77649E+14
SNORD29	-2,51124E+14
SAA4	-2,26661E+14
PNLDC1	-2,08259E+14
C11orf94	-2,04166E+14
CARD9	-1,98215E+14
SBSN	-1,97372E+14
FOXL2NB	-1,94827E+14
SLFNL1	-1,94686E+14
LTC4S	-1,85412E+14
C7orf61	-1,68945E+14
C1orf220	-1,5448E+14
ABCA12	-1,49177E+14
AKAP5	-1,32007E+14
NKAPP1	-1,2591E+14
MFSD7	-1,2565E+14
PLAC8L1	-1,2339E+14
TERT	-1,19723E+14
GRIN2C	-1,17271E+14
SNORA9	-1,13374E+14
BHMG1	-1,1158E+14
ZNF577	-1,09952E+14
SNORD45C	-1,08848E+14
SERPINA3	-1,08635E+14
COX16	-1,07392E+14
BCYRN1	-1,05077E+14
ADAM8	-1,02094E+14
DRD4	-1,00772E+14
HIST1H3B	-2,74164E+13
PTGDS	-1,673E+13
CYP24A1	-1,64803E+13
LOC103908605	-1,07604E+12
CCDC106	-0,970700418
ZGLP1	-0,970131094
UBAP1L	-0,939862967

SNORD36A	-0,934806384
FERMT3	-0,925623201
KRTAP3-1	-0,919502037
C8orf76	-0,910274566
ZNF586	-0,895944213
LOC100506472	-0,875723881
CTRL	-0,845975701
LOC100130950	-0,840921445
DLGAP1-AS2	-0,833860404
AGER	-0,831228444
YJEFN3	-0,824781323
LAT	-0,819261983
EAF2	-0,814340509
RBBP8NL	-0,801755447
SLC25A25-AS1	-0,785445569
IRF2	-0,783781235
ANKRD13B	-0,780339461
GNB3	-0,76236665
GAL3ST4	-0,755549437
TMEM145	-0,751039273
AOC2	-0,736854724
SLC22A20	-0,727292469
PYGM	-0,721746493
HSPE1	-0,719712363
CNFN	-0,719603083
TNNI2	-0,715789325
FCGBP	-0,674140129
QTRT1	-0,660904254
CIRBP-AS1	-0,660456221
MAPK15	-0,644956161
SLC27A5	-0,641802901
MSX1	-0,640162837
MYH3	-0,640111593
AMH	-0,627607353
DDX12P	-0,625230952
PRRT3-AS1	-0,624780793
FKBP11	-0,624390304
NPW	-0,624144544
ERVMER34-1	-0,611816709
PRSS30P	-0,606026257
CKMT1B	-0,599273105
TAF10	-0,588371622
DOK3	-0,585336934
MAMDC4	-0,583248895
TAF1A	-0,581677328
TMEM86B	-0,581257041
DANCR	-0,578839074

ADM5	-0,571365336
OBSL1	-0,561091493
CENPV	-0,550442894
NPR2	-0,549985278
SLC30A3	-0,549064289
RLTPR	-0,543138046
PADI3	-0,537863135
NME1	-0,536028617
COL7A1	-0,533793885
POLR3K	-0,53211419
NSUN5P1	-0,52991639
MCTS2P	-0,529677494
ZNF202	-0,529567518
APOO	-0,528129192
DUSP2	-0,524289764
KCND1	-0,523077935
PIDD1	-0,522231152
HSD11B2	-0,520634825
CHORDC1	-0,519816223
NUDT3	-0,513272671
ATP5G1	-0,510685109
MRM1	-0,507507932
PTGES	-0,505781295
GRAMD2	-0,503378363
RPIA	-0,502330752
DOC2A	-0,500777088
BRSK2	-0,498742494
FAM104B	-0,498290463
HAUS7	-0,497671837
ANKLE1	-0,492112761
NOP16	-0,491904288
C5orf45	-0,4917042
ASRGL1	-0,488387885
SNHG20	-0,488214201
FANK1	-0,487471272
KAT2A	-0,487238377
SNRPE	-0,481089448
TARBP1	-0,47743572
PABPC1L	-0,475895967
RAB26	-0,475089168
SRM	-0,47407459
CA5BP1	-0,473811143
CEBPD	-0,473293733
NPM3	-0,470943855
ATAD3B	-0,469979362
USP21	-0,463752838
LIAS	-0,462592193

TMEM97	-0,461814815
ITPKA	-0,461396644
ENGASE	-0,460487534
PIF1	-0,4588464
RRP8	-0,456807355
CCBL1	-0,456563683
THYN1	-0,454288184
MYC	-0,453392824
NENF	-0,448101773
NOP2	-0,447348548
ADAT3	-0,447256392
DNAJB1	-0,44685757
TRMU	-0,44646841
SNHG1	-0,445991463
TCOF1	-0,444616844
NAA16	-0,444325223
RELL2	-0,443068394
RSRP1	-0,442565215
EBPL	-0,438601202
SNRPD1	-0,438331151
TMEM216	-0,433877084
MRPS6	-0,431144455
TRMT1	-0,42795174
NOL12	-0,424226918
SERPINH1	-0,423467351
SSU72	-0,42176313
MANEAL	-0,421434146
C19orf44	-0,421211656
NDUFS5	-0,420855451
MARC1	-0,420713462
GAL	-0,41987383
BCCIP	-0,417989552
FAM86C2P	-0,414885997
MTERF3	-0,413257556
LUC7L	-0,412746601
ATP5D	-0,411596069
NOP56	-0,411501387
SLC5A6	-0,411060552
TMA7	-0,408327662
HEXDC	-0,405648425
BRIX1	-0,403566986
ZMYND19	-0,403349409
NSDHL	-0,402863144
GALNT14	-0,402192554
OMA1	-0,40153653
NCBP2-AS2	-0,400986139
SLC25A33	-0,400027239

APOBEC3F	-0,399451426
POLR1C	-0,398403418
NDUFA3	-0,396633983
EXOSC8	-0,396469589
TRIB1	-0,396184009
SIX5	-0,395607041
WASH1	-0,395416681
GPATCH4	-0,39533108
PRDM15	-0,395254027
WDR3	-0,395251308
TMEM99	-0,394550955
PFAS	-0,392973124
DDX11	-0,392925918
NOL11	-0,391684788
LYAR	-0,391203644
NXF1	-0,389836867
UTP14A	-0,389532755
KCNC4	-0,388863817
LRP3	-0,38851109
TBC1D30	-0,387789416
RBMXL1	-0,387082119
NAT9	-0,385925416
RBM14	-0,385650101
BCS1L	-0,38388934
BANP	-0,383471391
NEURL4	-0,379486238
UQCR10	-0,378556402
TRAP1	-0,378245912
RNMTL1	-0,376921214
DPH2	-0,376681636
SLIRP	-0,376369054
KIAA0907	-0,374552433
CHEK2	-0,374280431
TEFM	-0,374240103
TMEM39A	-0,374195148
CDK10	-0,371752955
MRPL11	-0,371467055
EED	-0,370944704
SRSF7	-0,36910516
RRS1	-0,368628171
EXOSC3	-0,367511877
STIP1	-0,365119057
ESF1	-0,365010077
CPNE7	-0,364732553
CD3EAP	-0,363793046
INTS9	-0,363663413
TXNL4A	-0,362527372

MRPL57	-0,361913221
KCNQ2	-0,361774558
EIF4A2	-0,361760709
FDFT1	-0,360133
RAD51C	-0,357975951
DDX31	-0,35756906
SNU13	-0,356894358
CLK2	-0,356442772
PNN	-0,356108406
FXN	-0,355709887
TONSL	-0,355680463
NGDN	-0,354201628
CTU2	-0,352317568
DHX37	-0,351827905
PRCC	-0,351504569
SNRPD2	-0,35117109
ORC5	-0,350771638
LDHB	-0,35001565
WRAP73	-0,349250123
EMC9	-0,349067376
LOC90784	-0,348197737
TRPM2	-0,346843957
MAT2A	-0,346520898
CCDC86	-0,345985872
PSMG1	-0,342940475
SNHG12	-0,342813476
PTRH2	-0,342426956
PRPF38B	-0,341768575
IGFBP2	-0,341735544
CNOT4	-0,3410138
MND1	-0,340582186
ZC3HC1	-0,339990411
HMGCS1	-0,339414689
INTS3	-0,339309347
HIBCH	-0,338291853
WDR74	-0,33727365
DESI1	-0,335171252
PRPF38A	-0,33419005
LUC7L3	-0,334173467
RABEPK	-0,33384134
PGAM5	-0,332777514
EIF3I	-0,33252813
CCNE1	-0,332180866
PINX1	-0,331504078
TBCCD1	-0,331253242
GNG5	-0,33068837
TIMM21	-0,330448996

NDUFA2	-0,329731574
RIOK2	-0,329703667
SPG21	-0,329422486
MALSU1	-0,328904409
JTB	-0,328784942
ALG6	-0,327852883
C1QBP	-0,327599901
NDUFAF2	-0,327332015
FDXR	-0,327250507
TIMM44	-0,325894586
PARP2	-0,325507402
NOP58	-0,32407512
C10orf2	-0,323042052
GLRX5	-0,322581763
NDUFAF4	-0,321841772
DNAAF2	-0,32168929
LYRM1	-0,320460559
SMO	-0,32040054
TOMM5	-0,319792991
ODC1	-0,319418201
PGP	-0,31912912
CCT8	-0,319046757
PA2G4	-0,318477488
DNAJA4	-0,318239769
ZNF146	-0,317785536
CEBPZ	-0,317209983
UQCR11	-0,317085884
GNL3	-0,315528159
NARFL	-0,315412229
ACAT2	-0,315229244
STX10	-0,315074985
PROSC	-0,314972019
WDR75	-0,314737022
CACYBP	-0,314625211
NOL6	-0,3143831
JMJD6	-0,314222872
IPO4	-0,311334368
SRRT	-0,310900726
DHCR7	-0,310899508
MICALL2	-0,310816768
IMP4	-0,310179689
DNAJC7	-0,310036867
SAMD1	-0,309053342
CCDC137	-0,308665632
THOP1	-0,307753884
ALKBH1	-0,307637059
TOMM40	-0,307214244

TUFM	-0,30698408
PPRC1	-0,306531399
MRPL18	-0,305339403
EIF5B	-0,305046749
CXXC1	-0,304519762
ONECUT3	-0,302887421
C11orf84	-0,301447857
CDC6	-0,301432422
STOML2	-0,300942237
HDDC2	-0,299996333
L3MBTL2	-0,299512929
ING5	-0,298516814
PRMT7	-0,298166432
EYA3	-0,297735951
RECQL4	-0,296885472
B4GALNT4	-0,296311508
PDCD11	-0,295677848
LIF	-0,294665165
KRI1	-0,294070597
NOP14	-0,293816177
FEN1	-0,292178043
RRP9	-0,291623231
SBK1	-0,291468283
CCT7	-0,289572905
NT5C3B	-0,289562419
NOL10	-0,289525816
C9orf114	-0,287571469
POLR3H	-0,286764406
KIAA0020	-0,286631795
NAT10	-0,286624468
EIF3L	-0,286153182
NIFK	-0,285874928
LRWD1	-0,284640628
UCK2	-0,284195297
SFT2D1	-0,283993697
YBX1	-0,283917844
ABCA7	-0,282556442
MRPS25	-0,282037754
DPH7	-0,280657108
UTP11L	-0,280572758
SSB	-0,279673282
GMNN	-0,279633331
LINC01234	-0,279082916
COPS5	-0,279049803
ADAM19	-0,278743597
CAD	-0,278003088
DHX30	-0,277532261

SRSF1	-0,277240418
ATG4B	-0,277224147
HSPA4	-0,276926654
QTRTD1	-0,27562368
CCAR2	-0,275603428
DNAAF5	-0,274752602
RUVBL1	-0,274115794
PDCD2	-0,273378191
NUDC	-0,273002687
CHAF1B	-0,273002496
NOC2L	-0,272747124
ACIN1	-0,270880814
SLC26A6	-0,270782346
GGCX	-0,270376034
NUDT9	-0,269715551
MRPS27	-0,269699753
FASN	-0,269567087
CWC27	-0,269345317
PRPF3	-0,26870036
PIM3	-0,268469781
MRRF	-0,268429593
RRP12	-0,268042968
IDI1	-0,267849368
ZC3H15	-0,267504262
MRPS22	-0,267285917
EIF3M	-0,266336747
EIF3E	-0,266305181
KARS	-0,266105648
QSOX2	-0,265023374
PTDSS2	-0,264727393
HSPA8	-0,2630223
TCP1	-0,262588964
RAE1	-0,262476636
HSP90AB1	-0,260986221
CCT4	-0,26025128
AUP1	-0,2586203
PCID2	-0,257886281
AEN	-0,257530533
BOD1	-0,257219506
SUPV3L1	-0,256303551
PPT1	-0,251205614
ALDH1B1	-0,250329822
YBX3	-0,250325351
BUD13	-0,24650496
RPAP1	-0,246110254
SF3B3	-0,245483071
ELAVL1	-0,240972607

POLR3D	-0,236840111
ZDHHC6	-0,236732126
GTPBP4	-0,23493437
SDCCAG3	-0,233968796
DNAJA3	-0,233257952
NCS1	-0,232824551
SRSF3	-0,231835446
HNRNPH3	-0,231437853
DNAJC11	-0,23062857
FARSB	-0,229193207
LEO1	-0,227964488
PALD1	-0,223599006
HSPA9	-0,2235798
CHTOP	-0,222013758
NUDT5	-0,221371551
USP19	-0,214312539
SDHD	-0,211272912
CCT2	-0,211231555
DUSP5	-0,20728355
LSG1	-0,206863613
CCT5	-0,200766357
NUP62	-0,198870232
CCT6A	-0,191280975
SNX5	-0,180153834

Upregulated

**Gene**	**log2FoldChange**
MEF2D	0,19414497
BCL2L13	0,20964503
TRAM2	0,21695979
OXSR1	0,22118065
KIAA1161	0,22728809
KCTD10	0,2277952
ITM2B	0,2286459
CAPN5	0,2442188
ZRANB1	0,250842
RGS3	0,26778879
LRRC16A	0,26979619
TK2	0,27067193
ALAD	0,27211703
DHX32	0,27362498
ELMSAN1	0,27588285
UNKL	0,27884197
IDS	0,28262424
HERPUD2	0,28306251
KIAA0513	0,28475205

MYO1D	0,28665381
SWAP70	0,28812815
CXorf38	0,29105453
RNF185	0,29606445
MXRA7	0,29613127
RASA3	0,29619564
SMARCA1	0,29701907
TMCO3	0,29709512
GRAMD3	0,29846902
FAM3C	0,30135206
SFXN3	0,30257936
ALDH3B1	0,30274681
HERC3	0,30392421
PQLC3	0,30561292
PDK1	0,30657925
RIPK4	0,31331316
TAP2	0,31394892
NIPAL3	0,31605936
SMAD7	0,31654188
VGLL4	0,31787209
USP43	0,31838032
DPYSL2	0,31878967
SERINC3	0,32000128
CAST	0,32113992
TMEM19	0,32219532
PPP2R3C	0,32508342
SHTN1	0,32544492
SCN5A	0,32715334
SNX33	0,32978427
PLEKHA2	0,33371221
MYO18A	0,33479739
STK17A	0,33538158
NCEH1	0,33677048
WAC-AS1	0,33731948
SLC35A5	0,34000486
SLC20A2	0,34017631
DDX58	0,34061536
REEP1	0,34095756
SPTBN1	0,34128115
KCTD9	0,34152348
ZNF185	0,34206735
TMEM87A	0,34277345
MACF1	0,3429262
MARCH8	0,34756776
SRD5A3	0,34801978
MFSD9	0,34948585
SLC1A1	0,3516059

ABHD2	0,35206418
L1CAM	0,35609474
DTX3L	0,35782693
FAM110C	0,35881059
KLF10	0,35919753
PAPSS2	0,35973195
ATF1	0,36375404
CCDC69	0,36414511
PREX1	0,36422554
CCDC82	0,36502084
MFSD6	0,36528904
LIMA1	0,36530001
DNAJC22	0,36874634
BACE1	0,37083871
ALS2	0,37396551
ZNF362	0,37482963
PROS1	0,37570915
B3GNT7	0,37576089
ZFYVE1	0,3765615
B2M	0,37679201
GNAZ	0,3819054
STYK1	0,38527658
ITGA3	0,38673804
TMEM87B	0,38678482
PRKCA	0,38684017
PQBP-1	0,38798102
HBP1	0,38825455
DNAJB5	0,38874167
UBXN2A	0,38949092
CAV2	0,39040415
SEPP1	0,3915233
MTMR10	0,39214929
RAB11FIP5	0,39252353
RELL1	0,39282997
TPPP	0,39316081
ITGB1	0,39628822
SLC24A1	0,39802865
OLFML2A	0,39896924
APH1B	0,39943059
BBS4	0,39950735
LAMB1	0,40281688
CYS1	0,40319524
PTP4A3	0,40473199
CCNG2	0,40475181
DTWD2	0,40602733
NDRG1	0,40752412
ZMYM5	0,4082403

KNDC1	0,40872455
PARP9	0,41028603
PI4K2B	0,41240862
SUSD1	0,41365634
RASSF2	0,41498601
DUSP10	0,41542135
RASSF5	0,41597414
CA13	0,41907982
CLUAP1	0,42112168
PPP1R3B	0,42192195
PNPLA8	0,42197784
XPA	0,42223689
LMO7	0,4255061
RPS6KA2	0,42591468
BCAP29	0,42617259
TC2N	0,43140821
SCAPER	0,4340962
RASSF4	0,43489116
SP110	0,43676086
YPEL5	0,43734126
GPR155	0,43818936
LAMP2	0,44185012
LZTS1	0,44195908
NAT1	0,44214121
CYP4V2	0,44543746
ARL6IP5	0,44694594
ZNF365	0,44811612
GSKIP	0,44863878
MCAM	0,44881173
ERMAP	0,44947842
PCDHGA10	0,44973224
KIF3C	0,45073302
SLC6A6	0,45205349
SLC26A2	0,45253088
GAB2	0,45283449
GGACT	0,45515684
ATL3	0,4564146
KIF13B	0,45704892
TESK2	0,45834809
GPR39	0,46107295
RAPGEF2	0,46137749
SYNPO	0,46185123
SLC25A16	0,4626291
DOK7	0,46400525
PLEKHF2	0,46895705
CLCN4	0,46895912
PTPN21	0,46915028

NUDT12	0,47250806
IL17RD	0,47265323
SLC41A2	0,47364905
ARL4C	0,47459226
CTSO	0,47546752
ACBD5	0,47643724
CDC42EP3	0,47684129
IL15	0,48136131
MYOM3	0,48230352
CLIP4	0,48266549
WDR66	0,48522301
NEK7	0,48529804
CPEB4	0,48566264
TP53INP1	0,48588888
ASAP2	0,49140472
PTTG1IP	0,49515526
C10orf32	0,49624591
ENPP5	0,49795651
ST6GALNAC2	0,49805023
SMARCA2	0,49898223
STX11	0,49923425
PCMTD1	0,50117193
F3	0,5016778
ZNF516	0,50286504
PTCH1	0,50302063
PSD4	0,50565787
SLC35E3	0,50855688
SMIM14	0,51534204
MYT1	0,51582205
ANTXR2	0,52056511
LPCAT2	0,52165192
PTPRN2	0,52435809
CREBRF	0,5251249
KIAA1217	0,52547589
GLIPR1	0,52597832
TANC2	0,52884422
CD24	0,53192057
MRC2	0,53278805
SOWAHB	0,53421114
GPR37	0,53720969
KLF12	0,53793134
KAT2B	0,53989225
GPR162	0,54612549
ELL2	0,55420548
ALCAM	0,55610067
SMPDL3A	0,55751349
KRCC1	0,55927878

ACVRL1	0,56058104
ARHGEF37	0,5609561
ABCA3	0,56544021
TIAM2	0,56557213
ST6GALNAC5	0,5657198
TMEM55A	0,56679297
SYNE3	0,569299
CCDC68	0,57004232
LIMCH1	0,57594281
WASL	0,57610688
PAQR8	0,58377882
CYP4F11	0,58913018
IRS2	0,59569627
RBM43	0,59888499
NATD1	0,6095357
GPRIN2	0,60974722
CSF1R	0,61432133
TENM3	0,61602275
FSCN2	0,61848003
SLC4A8	0,61871908
CD274	0,6208826
PPM1K	0,62647367
CLCN1	0,62730766
CYP4F3	0,63111083
CCNJL	0,63439343
MGC50722	0,63793632
PNMA2	0,64169695
ENC1	0,64436955
GOLGA8N	0,64728029
WDR72	0,64805585
TMEM56	0,64944716
TCAF2	0,64984656
MMRN2	0,65000832
MLLT3	0,65038906
PAG1	0,65557868
MICALCL	0,657025
SERPING1	0,65829288
BHLHE41	0,66082619
FZD7	0,66364273
KCNIP3	0,67118826
MAML3	0,67995771
REEP2	0,68120964
RUNX2	0,68356575
ZFYVE28	0,68760902
MMP28	0,69100361
GRIK2	0,77620171
EVI2B	1,0918E+14

GPR176	0,94355539
