# Supplementary material for: The synergism of SMC1A cohesin gene silencing and bevacizumab against colorectal cancer
Source: J Exp Clin Cancer Res. 2024 Feb 16;43:49. doi: 10.1186/s13046-024-02976-2 (PMC10870497; doi:10.1186/s13046-024-02976-2)
Supplement: Supplementary file 10 — Additional file 10: Table S5. Dysregulated genes (down- and upregulated) following bevacizumab treatment. [file 13046_2024_2976_MOESM10_ESM.pdf]

Table S5. Dysregulated genes (down- and upregulated) following bevacizumab treatment.

Downregulated

Gene	log2FoldChange
CSF3	-5,91239E+14
PLA2G4E	-5,69514E+14
S100A9	-5,56181E+14
IL17C	-5,2276E+14
CFTR	-5,08472E+14
IL1B	-5,00805E+14
LRRC55	-4,8999E+14
C1orf127	-4,66273E+14
PIK3R6	-4,51555E+14
MGC32805	-4,39935E+14
SELENBP1	-4,38274E+14
SPRR2A	-4,30409E+14
IL22RA2	-4,24646E+14
PAEP	-4,13194E+14
SPRR2D	-3,93983E+14
ADGRG3	-3,92451E+14
GP6	-3,91603E+14
SNCAIP	-3,91161E+14
C15orf48	-3,85162E+14
LTB	-3,65827E+14
PI3	-3,57983E+14
S100P	-3,50914E+14
GRHL3	-3,44101E+14
LTA	-3,34144E+14
LEAP2	-3,0653E+14
WFDC21P	-2,97808E+14
KYNU	-2,89739E+14
PABPN1L	-2,80307E+14
PILRA	-2,79644E+14
BDKRB1	-2,78535E+14
GNRH2	-2,75399E+14
CIITA	-2,70594E+14
ZNF763	-2,67019E+14
CD74	-2,51711E+14
JAKMIP2-AS1	-2,4679E+14
CYP24A1	-2,4626E+14
SAA1	-2,43801E+14
FOXL2NB	-2,33138E+14
ASB2	-2,31141E+14
SAA2	-2,27877E+14
ADGRF1	-2,20135E+14
MEFV	-2,14204E+14
KRT23	-2,1E+14

CYP3A5	-2,08278E+14
CDIPT-AS1	-2,08036E+14
CFB	-2,04355E+14
TWIST2	-1,96559E+14
SNORD28	-1,90317E+14
TNFSF14	-1,90033E+14
KLRC3	-1,88629E+14
ANKRD31	-1,87561E+14
LRG1	-1,86403E+14
CXCL8	-1,85685E+14
ZC3H12A	-1,84218E+14
SENP3-EIF4A1	-1,81689E+14
CCL28	-1,8056E+14
SBSN	-1,79326E+14
CPAMD8	-1,78504E+14
RTCA-AS1	-1,72584E+14
C2orf66	-1,70634E+14
POM121L9P	-1,70133E+14
OTUD7A	-1,64869E+14
VNN1	-1,64137E+14
KLRC2	-1,59436E+14
CARD9	-1,59138E+14
NCF4	-1,55592E+14
SMIM22	-1,52201E+14
C5	-1,51152E+14
LYZ	-1,4694E+14
ABCA12	-1,44923E+14
NFKBIZ	-1,44726E+14
IL32	-1,41491E+14
GBP2	-1,4146E+14
C2	-1,41389E+14
FAXDC2	-1,39072E+14
LOC100506124	-1,38803E+14
SSSCA1-AS1	-1,38357E+14
LOC100506472	-1,32533E+14
SOD2	-1,31909E+14
SNORA9	-1,28784E+14
SPATA6L	-1,27352E+14
LY6D	-1,26337E+14
SEMA4A	-1,26161E+14
NTRK1	-1,22989E+14
ALDH1A3	-1,22572E+14
SLC16A6	-1,21575E+14
LOC102723927	-1,2123E+14
TMPRSS13	-1,18901E+14
GPM6B	-1,18781E+14
SNORD14E	-1,18571E+14

ROCK1P1	-1,18255E+14
LOC100130705	-1,13146E+14
ZNF577	-1,1191E+14
UBAP1L	-1,09456E+14
LINC01468	-1,0717E+14
TNFAIP2	-1,06099E+14
TEX14	-1,0529E+14
KRTAP3-1	-1,02532E+14
GRIN2C	-1,0231E+14
FCGBP	-1,00793E+14
ACRBP	-1,00786E+14
TNFAIP6	-4,43629E+13
MIR3189	-4,0931E+13
C10orf62	-3,99083E+13
C1QTNF1-AS1	-3,66738E+13
GSAP	-2,33409E+13
RASD1	-2,06698E+13
EHF	-1,99866E+13
PPAP2B	-1,7555E+13
SNORD55	-1,30639E+12
AKAP5	-0,996643118
MAP3K8	-0,994331262
DNAH17	-0,991613434
BASP1	-0,978795494
ADAM8	-0,977621989
ADGRV1	-0,968620309
NAPSA	-0,943166709
LOC389602	-0,942713574
NOD2	-0,934084826
CASP10	-0,927868123
ZGLP1	-0,924438581
GDF15	-0,913600025
ATAD3C	-0,898562124
FBXL19-AS1	-0,897859058
GPR35	-0,889540015
PLIN4	-0,874521155
YJEFN3	-0,866988414
TFPI	-0,864661237
LOC101929125	-0,864287897
SAT1	-0,857596179
GSTM2	-0,857236885
LOC101929709	-0,84972993
CEACAM19	-0,835098482
NPIPB15	-0,829122141
GNB3	-0,811726286
SCARF1	-0,801885763
AGER	-0,8002498

MST1P2	-0,797118905
IFNGR1	-0,794457065
MYH3	-0,79182765
NLGN3	-0,775983091
NR4A2	-0,774368479
SPIN2B	-0,763761835
LTB4R	-0,755084632
DDN	-0,74469673
C9orf16	-0,740424787
CSTB	-0,733599858
OGDHL	-0,723429277
GDPD3	-0,720523996
APOO	-0,708619558
LINC01012	-0,706281765
LOC100133091	-0,704462732
ZC3H12C	-0,70408092
CSAD	-0,696180845
SEMA6A	-0,686283579
ZDHHC11	-0,684436649
SNHG10	-0,681426966
LUCAT1	-0,678658396
PRKXP1	-0,6728843
LINC01089	-0,672655163
TMEM145	-0,668972894
NFATC4	-0,66008135
DUSP16	-0,658597247
SCO2	-0,656000077
TMEM132A	-0,651295845
DNAH17-AS1	-0,648169709
SLC11A2	-0,644511706
CFAP53	-0,642084207
RAPGEFL1	-0,641747302
NAA16	-0,63864455
LOC613037	-0,632645161
QTRT1	-0,632391395
AHSA2	-0,631470552
RHBDL1	-0,63143341
OVGP1	-0,626858198
SLC12A2	-0,625754056
SLC43A2	-0,625442624
ZNF692	-0,620398226
RELL2	-0,618426588
RNF207	-0,618219236
MAPK15	-0,615577483
CCAT1	-0,614926623
TNFAIP3	-0,607957721
RRAD	-0,603211751

RSRP1	-0,584405238
PPARGC1B	-0,581902853
SEC31B	-0,579447923
AIFM3	-0,572478288
PRDM15	-0,571808301
COL7A1	-0,570343643
RPL32P3	-0,569491007
ARV1	-0,562317096
DUSP2	-0,561880785
NPR2	-0,561249513
CKMT1B	-0,561245984
TARBP1	-0,560966635
TRAF1	-0,559884033
PABPC1L	-0,558740019
IGSF9	-0,556291196
FAM227A	-0,555873934
NFIA	-0,55576979
TFRC	-0,554170078
ASNS	-0,552517686
SNHG3	-0,552502178
SCNN1D	-0,549518682
ADGRE2	-0,544587053
AREG	-0,527174007
AMH	-0,522922765
STK19	-0,522359904
ZNF239	-0,516942806
NSUN5P2	-0,515279417
TMEM99	-0,510556503
VDR	-0,508971686
TRMT11	-0,50799819
KLHL17	-0,507512617
ATP6V1C2	-0,50572016
DDX12P	-0,503753896
EREG	-0,503489009
CLDN15	-0,503407821
TMEM198B	-0,501997237
RIT1	-0,501891979
KANSL1L	-0,501040768
SNHG4	-0,498444554
TRPV1	-0,49781502
DNHD1	-0,497586177
ANKRD19P	-0,49610187
RBMXL1	-0,493328626
NIPBL-AS1	-0,493323209
LYN	-0,492428653
BBC3	-0,491956836
CXCL16	-0,490232957

FOXD4	-0,49020235
OR51B5	-0,486693479
LYRM1	-0,481393185
SLC25A37	-0,480835933
PAXIP1-AS1	-0,475305089
KCNK5	-0,473790218
KLHL15	-0,467638191
CPNE7	-0,465636349
ZNF202	-0,462996943
C5orf45	-0,457832882
MYC	-0,453122875
FDXACB1	-0,449850965
GUSBP11	-0,449096133
MTRR	-0,44842949
SLCO4A1	-0,44826565
LDHB	-0,445447243
CYP27B1	-0,438314782
HOXC9	-0,435778688
GOLGA8B	-0,430493953
PP7080	-0,42992198
PCBP1-AS1	-0,429696073
LIAS	-0,429464172
GTF2IP20	-0,429258095
DRAM1	-0,425546948
RNPC3	-0,420421544
SYS1	-0,41975208
NFKB2	-0,418650931
POLR1B	-0,417614973
ATP5G1	-0,417311854
PIDD1	-0,413413554
SLC4A11	-0,412521008
AHI1	-0,411551972
RPL11	-0,411440475
RPIA	-0,407329739
DNAH14	-0,406050384
FKBP14	-0,405869975
HERC2P2	-0,405365996
LINC00641	-0,405353333
RLTPR	-0,401602996
FAM206A	-0,399532362
CLK4	-0,398306419
USP21	-0,396791736
RILPL2	-0,395016669
WDR3	-0,39462511
ZNF276	-0,391146966
C10orf2	-0,390515866
SLC18B1	-0,389955809

ESF1	-0,389698608
DHX34	-0,389342684
PRPF38B	-0,388096466
SNHG1	-0,388033814
SNAPC4	-0,387755775
PCCA	-0,387367934
RRP8	-0,385557793
FAM155B	-0,38550039
GDAP1	-0,385287102
CCDC57	-0,385261615
TBPL1	-0,384031728
PHF14	-0,381229222
BTG2	-0,380852439
PLXNB1	-0,379545701
TJP3	-0,378968053
PDXDC2P	-0,378865226
NOP16	-0,375254196
LETMD1	-0,374968313
DNAAF2	-0,374853469
PSTPIP2	-0,374018904
APOBEC3F	-0,373450953
DFFB	-0,373015337
DCUN1D3	-0,371695246
KPTN	-0,369180681
PUS1	-0,367420014
HEXDC	-0,366796536
NAT9	-0,366593522
PDE7A	-0,365668662
PUS3	-0,36553163
EBPL	-0,365150158
KAT2A	-0,36287739
WRAP73	-0,361497505
NFKB1	-0,360762849
RPAIN	-0,360293322
ORC5	-0,36013104
HSPD1	-0,357405255
RPS25	-0,356562117
MIR100HG	-0,355175984
TXNL4B	-0,355119713
ALKBH1	-0,354233192
SLC17A9	-0,353752348
UFC1	-0,352971374
CLK2	-0,352581101
ZNF271P	-0,352490749
TMEM39A	-0,350599521
DNAJC2	-0,349106782
SKIV2L	-0,345803074

RIOK2	-0,345691772
KCNQ2	-0,345146292
C1orf109	-0,344817969
NOP2	-0,343442024
SYT7	-0,343438526
MARC1	-0,343342036
C9orf91	-0,342917612
NKRF	-0,341931076
USF1	-0,34145022
MOGS	-0,341229528
GCH1	-0,339087215
C1orf52	-0,336299045
NEURL4	-0,332442063
DDX10	-0,332210836
SFXN4	-0,330832922
MRRF	-0,328650734
ADAT2	-0,326779609
PNN	-0,325083577
NXF1	-0,324437098
GUF1	-0,323213018
LOC90784	-0,322585507
C3orf62	-0,319439655
HDDC2	-0,317961051
MRPS25	-0,315672502
MTERF3	-0,313188222
BCCIP	-0,312154246
CBWD5	-0,308686716
MARS2	-0,307880338
ARL14EP	-0,307296685
SNRPE	-0,30560956
THYN1	-0,304242244
NOL6	-0,300379713
NOL10	-0,297980961
NFKBIB	-0,297252177
PFAS	-0,29709256
ACTR5	-0,296540466
TRAP1	-0,295587063
E2F4	-0,295247387
ING5	-0,292499657
COA4	-0,292122977
MAP2K3	-0,289752704
RPS18	-0,288393694
LOC100506548	-0,288145332
KIAA1147	-0,287797492
MCCC1	-0,287481401
STX16	-0,286966118
NARFL	-0,285868122

CYB5D1	-0,285094908
WDR75	-0,284805797
NDUFS5	-0,283370424
GBA2	-0,282862646
INTS10	-0,27931461
MAT2A	-0,278593888
SLC5A6	-0,277149454
SPATA2	-0,276850962
DDX31	-0,276533957
QSOX2	-0,27591126
ZNF384	-0,275374748
RIOK1	-0,275314992
MAP3K13	-0,275129431
SLC35E2B	-0,274922628
ZMYND8	-0,272448381
FASTKD5	-0,267052641
PPP1R10	-0,266398107
ZDHHC6	-0,264170865
CNPY2	-0,258386411
EIF3I	-0,258142793
ZNF251	-0,258139733
MTIF2	-0,256968223
PRCC	-0,256009247
RAP1GAP2	-0,255812782
VPS45	-0,25175989
ZNF131	-0,250281214
ORC2	-0,245358972
MCCC2	-0,238176324
SDHD	-0,227445097
PNISR	-0,224383571
OGT	-0,215733928
ZNF317	-0,201884023
RBM25	-0,189399799
CCNL1	-0,184729885

Upregulated

**Gene**	**log2FoldChange**
ZNF146	0,111017115
DNAJC5	0,186629514
ARPC5	0,189119697
GNB1	0,196360402
IDS	0,197082191
SEPT9	0,202739677
ILK	0,207375849
CALM1	0,208475082
DCAF6	0,210991088

PRMT2	0,212379539
PRPSAP1	0,215865234
USP4	0,217857283
ADD1	0,217956411
MAD1L1	0,220450921
WDR1	0,223963425
PLS3	0,227490185
TPM3	0,232135565
HERPUD2	0,233027494
CLIC1	0,233242119
NCK2	0,233950802
KCTD5	0,234391807
SH3BP5L	0,235277592
FNBP1	0,235770555
TMCC1	0,238048314
SDC3	0,238568822
ZFYVE21	0,239278668
TRIP10	0,239518236
MAP4	0,242800165
TRAM2	0,243821376
OXSR1	0,245993415
PCYT1A	0,24648055
TUBB6	0,246622969
NAGA	0,247350052
PITRM1	0,249994436
C12orf75	0,255100827
MAPKAPK3	0,255808066
KIF1C	0,256830947
PKM	0,258221937
RASA3	0,258914058
SRD5A1	0,261308906
ATP6V1E1	0,262411562
MSN	0,262512012
TPGS2	0,264349667
RHEB	0,266298007
EZR	0,266760914
STXBP1	0,267986252
SHANK3	0,268985955
DRAP1	0,269159476
SHTN1	0,269237582
SPSB1	0,271108914
PREP	0,275314374
KIAA1161	0,276985204
INF2	0,279231021
CD9	0,28066067
RHOC	0,281510944
EIF4EBP1	0,281808445

GNAI2	0,283235235
MYL12A	0,284823558
ITGB5	0,285331514
SKA2	0,28540712
BCL2L1	0,286942888
FAM3C	0,287359476
GNB5	0,289381925
MSANTD3	0,290514078
ARPIN	0,290655713
GLUL	0,293851745
ANXA2	0,294481548
DBNL	0,294484805
RTN4	0,295462162
TMEM19	0,296258725
MYO1D	0,297519489
CTSV	0,298230104
KLF6	0,298621993
ARL6IP5	0,301392854
SUN1	0,301642371
KLC1	0,302118626
BAIAP2	0,310273638
GPC1	0,312230543
CPNE2	0,312685402
RAC2	0,314578554
CYTH3	0,315199921
RAP1GDS1	0,31572497
DMTN	0,316099787
TMEM55A	0,316878886
CALM2	0,317532816
MVP	0,317664117
TMEM87A	0,318610777
ITPR3	0,318932189
SYDE1	0,320216026
MTL5	0,32079582
CHST7	0,321794443
PACSIN2	0,321880276
EMP3	0,321942001
INADL	0,322877476
CAST	0,324582609
DNAJC22	0,324798533
PRRG4	0,330453727
BAIAP3	0,331152154
FAM64A	0,331784155
PARVA	0,332190369
TES	0,333521446
GSN	0,33421221
CAV2	0,334335848

VGLL4	0,33507229
KRT8	0,33529587
ADGRE5	0,336101998
PCTP	0,336432082
SKAP1	0,33648612
UBXN2A	0,337382962
PPP1R26	0,337557218
NFIX	0,341335298
ABHD2	0,348683462
PQLC3	0,348997981
GPR161	0,349397853
AIG1	0,353650382
CD99	0,353929671
LINC00152	0,353964774
PBX3	0,356177422
PXDC1	0,356273931
FXYD5	0,356753762
COL6A2	0,357079751
TCP11L1	0,357386523
NOP14-AS1	0,359373409
TMEM245	0,360540652
TGFA	0,360813488
PLA2G15	0,360879002
C11orf68	0,362495965
PLEC	0,362797253
RNF115	0,363105987
SHB	0,363250959
SPTBN1	0,365351957
LTBP2	0,365550484
ZNF385A	0,366457492
GAS2L1	0,369459989
SLC35E4	0,369712052
MXRA7	0,370245371
HES2	0,370424275
TLDC1	0,371513804
TUBB4A	0,371971504
CTH	0,372801207
PROSER2	0,372848089
LRRC8A	0,375708879
ENOX1	0,377046419
SRPX	0,377560507
MYADM	0,377640254
RAB11FIP5	0,380675227
SMAD7	0,382092518
ARHGAP21	0,384299928
MPP1	0,384559745
COCH	0,385336772

MYO18A	0,385910598
PHTF1	0,386391314
SRRM3	0,388649361
SEC14L1	0,388971603
PIM1	0,388978413
MICALL1	0,389290036
PLEKHA6	0,390361695
KLC3	0,395256593
LASP1	0,396050709
CHRD	0,396435297
KIF13B	0,397689739
LMNA	0,401653205
LIMCH1	0,402783682
ALS2	0,40709087
RRBP1	0,407457013
SH3BP4	0,407681055
TTC7A	0,409597138
CDKN2B	0,410507204
SNX25	0,411342414
RASA1	0,413035434
GLIS3	0,414463899
ESPN	0,41509297
GGACT	0,41537295
FLNA	0,415829173
BCAR3	0,415892359
ERBB2IP	0,416235779
LATS2	0,417865533
TPPP	0,420523621
SHANK2	0,427074279
RIPK4	0,430918252
PMP22	0,431344299
ATL3	0,431694688
OSBPL10	0,434533822
CRIP1	0,435949513
CTDSPL	0,436158921
NES	0,437038199
TANC2	0,438639824
NDRG1	0,441411606
CMTM7	0,446294713
TRIM6	0,447369101
LRRC37B	0,447493111
C1orf116	0,448736198
SLC44A3	0,449214467
SERPINB5	0,45271562
ALDH3B1	0,453708197
NWD1	0,45411053
KNDC1	0,454802098

EBF4	0,454983477
TMEM107	0,456750518
MYH9	0,458719727
ZNF185	0,459874254
RGS14	0,461275374
AMOTL2	0,46129003
SRD5A3	0,465815422
TUBA1A	0,466998638
KIF12	0,467212907
UBALD2	0,467675975
PAPSS2	0,467980848
MLPH	0,468403503
CUL4B	0,474620606
RGMA	0,47690837
AAMDC	0,477340504
FGFR3	0,477479361
LPCAT2	0,477496837
COL13A1	0,478869499
S100A10	0,478925116
L1CAM	0,479094919
ELL2	0,479625458
TGFBR1	0,479864784
AMDHD1	0,486266996
TIMP2	0,487630045
ZNF680	0,48784239
FHL1	0,487852065
CDKN2D	0,487915366
PRKCA	0,492919472
CCDC68	0,493754377
CDC42BPA	0,494881397
GOLIM4	0,499258342
CLIC3	0,499826597
KHDC1L	0,5043815
PDGFC	0,506547327
SYNPO	0,506570504
UACA	0,50885558
REEP1	0,510596056
PTTG1IP	0,51121399
ENDOD1	0,51229827
LGALS1	0,518495485
LIMS2	0,526290876
FGD1	0,528055832
WDR66	0,529005889
IL17RD	0,531237463
CSPG4	0,533380027
COL12A1	0,534139093
OGFRL1	0,536168301

ARHGEF26	0,539151091
ABCA3	0,539664176
HTR1D	0,539817935
HR	0,540326455
TBC1D2	0,540419536
OLFML2B	0,541238837
STIM1	0,542288611
SH3TC2	0,543938195
PTPRN2	0,54436926
RASSF6	0,545884689
GAS6	0,547206748
GAB2	0,547846896
RASSF2	0,54790959
OLFML2A	0,556560069
CTNNAL1	0,556726669
CLIP4	0,557189415
CD22	0,558765881
ST6GALNAC2	0,560699018
HCFC1R1	0,560826846
LYPD6	0,565276924
CNN3	0,567486569
PDLIM2	0,571294537
C1QL4	0,584921015
ALCAM	0,586531408
PADI2	0,58961707
MAP2	0,5898618
ABLIM3	0,592873534
SMARCA2	0,59291364
CRIP2	0,593391616
STARD5	0,594820517
TMEM173	0,595959661
GLI2	0,598851426
ANXA2P2	0,599643602
CDC42EP3	0,60178134
PRICKLE1	0,611753015
SYNC	0,614271956
FAM46C	0,615962397
ZFYVE28	0,616745519
TINCR	0,618122915
CLCN1	0,622289526
PLK2	0,625698715
BVES	0,626338317
ARL4D	0,630327137
PRKCDBP	0,638770738
ANTXR2	0,645682183
KAT2B	0,645776784
FKBP1B	0,64709175

TMEM255B	0,650139991
AFAP1L1	0,65204678
TAB3	0,659505291
TGFB1I1	0,660707673
LOC100507002	0,668038508
ASAP2	0,670462583
BHLHE40-AS1	0,691569906
MGLL	0,694642694
CGB8	0,695619144
GNG13	0,703584181
ROR1	0,707061328
USH1G	0,710083213
LOC102724279	0,727295203
CCNJL	0,733235601
TIAM2	0,740712539
ECM1	0,74967465
MBNL3	0,75143721
MCAM	0,755805567
TENM3	0,756956655
TLN2	0,759387826
ITGB4	0,766731937
CST6	0,770852971
SUSD4	0,771035054
LYPD1	0,772051159
S100A2	0,774745453
YBX2	0,790131808
NTN4	0,791937484
FAM129A	0,796877862
NEDD9	0,79811786
ST6GALNAC5	0,800114141
CSF1R	0,801671826
NOS1AP	0,802395259
RGS9	0,811531978
INHBB	0,840231196
GPR176	0,844753831
TMEM200A	0,853734888
SLC9A9	0,899910011
FBP1	0,901127619
EVI2B	0,902948722
DLK2	0,913400796
ENC1	0,914228611
NKX3-2	0,92346269
SERPINE1	0,933604272
JMJD1C-AS1	0,953589348
HEG1	0,971801843
DENND2A	1,25248E+12
GRIK2	1,07244E+13

KRT86	1,55485E+13
TCN2	1,00047E+14
CYP4F11	1,00848E+14
RUNX2	1,08511E+14
COL15A1	1,08868E+14
MAMDC2	1,14689E+14
WNT5B	1,17133E+14
LCTL	1,17682E+14
PI16	1,18279E+14
EMILIN1	1,24787E+14
SERTAD4	1,3839E+14
CADM1	1,44702E+14
PRSS23	1,48775E+14
CTSE	1,63138E+14
CXCR3	1,70358E+14
KRT81	1,72713E+14
LINC01605	1,97619E+14
SND1-IT1	2,71459E+14
