# Supplementary material for: The synergism of SMC1A cohesin gene silencing and bevacizumab against colorectal cancer
Source: J Exp Clin Cancer Res. 2024 Feb 16;43:49. doi: 10.1186/s13046-024-02976-2 (PMC10870497; doi:10.1186/s13046-024-02976-2)
Supplement: Supplementary file 11 — Additional file 11: Table S6. Common dysregulated genes between PRJNA635121 bioproject and our bevacizumab-treated tumors. [file 13046_2024_2976_MOESM11_ESM.pdf]

Table S6. Common dysregulated genes between PRJNA635121 bioproject and our bevacizumab-treated tumors.

Gene	PRJNA635121 (log2FoldChange)	This work (log2FoldChange)
SAA2	-2,9891E+14	-2,27877E+14
SAA1	-2,5861E+14	-2,43801E+14
HTR1D	-1,9803E+14	0,539817935
FAM206A	-0,60420457	-0,399532362
PDLIM2	0,710350376	0,571294537
CLIP4	0,791467406	0,557189415
RASSF2	0,812101076	0,54790959
NFATC4	0,920511086	-0,66008135
PILRA	0,948482232	-2,79644E+14
CHRD	0,980201032	0,396435297
INHBB	0,987665463	0,840231196
EBF4	1,0333E+14	0,454983477
SLC16A6	1,03884E+14	-1,21575E+14
LTB	1,15961E+14	-3,65827E+14
GSTM2	1,26209E+14	-0,857236885
POM121L9P	1,37693E+14	-1,70133E+14
