# Supplementary material for: The synergism of SMC1A cohesin gene silencing and bevacizumab against colorectal cancer
Source: J Exp Clin Cancer Res. 2024 Feb 16;43:49. doi: 10.1186/s13046-024-02976-2 (PMC10870497; doi:10.1186/s13046-024-02976-2)
Supplement: Supplementary file 12 — Additional file 12: Table S7. Dysregulated genes (down- and upregulated) following combo treatment. [file 13046_2024_2976_MOESM12_ESM.pdf]

Table S7. Dysregulated genes (down- and upregulated) following bevacizumab plus shRNA treatment.

Downregulated

Gene	log2FoldChange
S100A9	-5,02029E+14
CFTR	-4,97802E+14
LINC01126	-4,9728E+14
SPRR2E	-4,83884E+14
PLA2G4E	-4,66298E+14
IL1B	-4,65491E+14
CRABP1	-4,27273E+14
ALK	-4,23782E+14
SPRR2A	-4,23471E+14
C1orf127	-4,2285E+14
AANAT	-4,18288E+14
PLA2R1	-4,09692E+14
ZNF606	-4,05375E+14
RNF148	-4,03043E+14
LRRC55	-3,89021E+14
C15orf48	-3,73878E+14
LTA	-3,54182E+14
SFTPD	-3,503E+14
EDAR	-3,43388E+14
NRG1	-3,41039E+14
BNC2	-3,397E+14
TNFAIP6	-3,2551E+14
PI3	-3,17218E+14
SMIM10	-3,0744E+14
KY	-2,97464E+14
HIST1H3B	-2,79615E+14
S100P	-2,7551E+14
PDZRN3	-2,75112E+14
CIITA	-2,73797E+14
PKNOX2	-2,72102E+14
LOX	-2,6661E+14
AFAP1-AS1	-2,64813E+14
PCDH12	-2,61366E+14
GREM2	-2,59158E+14
MARCH1	-2,54907E+14
SULF1	-2,53112E+14
PRKCB	-2,50392E+14
KRT23	-2,47464E+14
SAA2	-2,35959E+14
AEBP1	-2,32071E+14
LOC101928100	-2,20024E+14
DNAAF1	-2,09085E+14

ADGRF1	-2,06112E+14
ALDH3B2	-1,99459E+14
SFRP1	-1,95702E+14
PILRA	-1,94482E+14
CYP3A5	-1,93702E+14
SBSN	-1,90825E+14
ABCA1	-1,90513E+14
COL3A1	-1,84462E+14
NOVA1	-1,8029E+14
ASB2	-1,76235E+14
CPAMD8	-1,74608E+14
SIRPB2	-1,72641E+14
UNC13A	-1,68813E+14
CARD9	-1,65686E+14
ABCA12	-1,65653E+14
ZEB2	-1,65046E+14
DDR2	-1,6388E+14
FAM83A	-1,63204E+14
DMD	-1,58937E+14
CXCL8	-1,55015E+14
SLPI	-1,54906E+14
KLHDC7B	-1,54516E+14
CELF2	-1,53472E+14
ELMO1	-1,51332E+14
GRIA3	-1,4957E+14
RUNX1T1	-1,4601E+14
TBX15	-1,4518E+14
KCNQ5	-1,33678E+14
C2	-1,32041E+14
CASP1	-1,30809E+14
PLCG1-AS1	-1,29896E+14
POM121L9P	-1,29664E+14
KLRC3	-1,29073E+14
SPATA6L	-1,28005E+14
KLRC2	-1,27545E+14
SNORD55	-1,27326E+14
FGF2	-1,27103E+14
AMN	-1,2586E+14
XDH	-1,25792E+14
ROS1	-1,2543E+14
POU2F2	-1,2456E+14
BMP5	-1,22494E+14
CSGALNACT1	-1,21636E+14
LY6D	-1,21295E+14
NCR3LG1	-1,20722E+14
PTPRB	-1,158E+14
BASP1	-1,15531E+14

SNORA9	-1,1532E+14
CXCL14	-1,13432E+14
FILIP1L	-1,1204E+14
POF1B	-1,10791E+14
AKT3	-1,09233E+14
MMP2	-1,09052E+14
SLC16A6	-1,0847E+14
TAPBPL	-1,07687E+14
SNORD74	-1,06796E+14
PTENP1	-1,0568E+14
EBLN2	-1,05509E+14
GRIN2C	-1,04937E+14
GPR35	-1,04852E+14
TCF4	-1,04532E+14
LOC389602	-1,03018E+14
FHDC1	-1,02289E+14
LINC00944	-1,01808E+14
LINC01397	-3,93422E+13
WFDC21P	-2,92563E+13
SFRP2	-2,45747E+13
RASD1	-2,42321E+13
TRPS1	-1,9562E+13
TNFSF14	-1,66474E+13
IL32	-1,65112E+13
CDSN	-1,38317E+13
C3	-1,38207E+13
KCNK12	-1,27982E+13
C1QTNF1	-1,26187E+13
EPS8L3	-1,25882E+13
TMPRSS13	-1,05779E+13
IL17C	-43512380532
AZGP1	-0,975679062
TERT	-0,961879622
TRIM22	-0,9437211
ADGRV1	-0,934687782
IL23A	-0,933821468
HAL	-0,925877063
SGMS1-AS1	-0,918280891
SGK1	-0,915223829
GPR158	-0,906955945
NKX1-2	-0,898913085
FST	-0,875106655
HLA-DPA1	-0,873117803
BHMG1	-0,868743117
NCKAP1L	-0,868637894
IRF2	-0,859968629
TNFAIP3	-0,857705082

NAV3	-0,851290034
FCGBP	-0,847188994
DLGAP1-AS2	-0,843425437
LOC101928279	-0,840975071
MARCH3	-0,814999349
FTH1	-0,804273034
RBBP8NL	-0,802647152
BSN	-0,789656326
DIAPH2	-0,787345628
MAFB	-0,783474946
QKI	-0,756791835
FN1	-0,74981229
ICAM1	-0,74734029
ICOSLG	-0,74287242
DNAH5	-0,733786011
FOXP1	-0,728137382
BACH1	-0,725069541
LOC100288181	-0,717299421
AHCYL2	-0,716966858
CEBPD	-0,715038032
GALNT14	-0,703378743
SLCO3A1	-0,703000467
RMRP	-0,700604722
PRKACB	-0,700406278
NPHS1	-0,699192179
EBF1	-0,692776819
LOC100133091	-0,692597227
ITGA2	-0,686971381
KANSL1L	-0,686468549
DUSP16	-0,681005153
SLC43A2	-0,678282735
DDI2	-0,676145919
ALOX5	-0,673611886
MT1X	-0,667365497
FBXL19-AS1	-0,664075608
NCOA7	-0,644217423
SEMA6A	-0,623410088
EXT1	-0,622892418
NR4A2	-0,622031998
DDX60L	-0,610183968
CYP27B1	-0,591743917
PVRL4	-0,58543974
OGDHL	-0,584449016
RAD54L2	-0,577046508
TGFBR3	-0,57571949
ELK3	-0,57404786
CECR7	-0,573907129

DTNA	-0,573315192
CSTB	-0,571631582
RC3H2	-0,567478561
DSE	-0,565735797
KSR1	-0,559877561
ZAK	-0,557241989
ASXL2	-0,554846843
CD82	-0,554822486
RRAD	-0,5540181
SORBS1	-0,552590486
GRAMD2	-0,549179088
DOC2B	-0,547796373
MFSD2A	-0,546897568
OR51B4	-0,545878768
SLC9A5	-0,545426222
DNAH17-AS1	-0,542129611
AREG	-0,541178443
TARBP1	-0,520331571
C1RL-AS1	-0,517782526
HYAL1	-0,511521559
LRRC8B	-0,508569529
SLC16A9	-0,508001082
BRSK2	-0,507351102
EFNA1	-0,50685317
CXCL16	-0,506474164
RFX7	-0,504995335
CRACR2A	-0,503007818
LYRM1	-0,502151265
KLHL15	-0,501143754
SEMA3C	-0,498602325
PLAUR	-0,497872393
GRHL1	-0,496658915
VPS9D1-AS1	-0,49610435
MREG	-0,493244602
FAM208B	-0,483542763
PSD3	-0,482509388
KCNC4	-0,477920206
C11orf30	-0,477368717
IQCD	-0,476860575
DDN	-0,474691512
SNX13	-0,473411685
VDR	-0,470620657
SESTD1	-0,467779147
MCOLN2	-0,467667564
SLC39A8	-0,466848159
PIAS1	-0,465938132
IGSF9	-0,465897151

MSI2	-0,465554076
WNK2	-0,462241617
MAFG-AS1	-0,46183392
BBC3	-0,461535139
DMXL2	-0,459809689
RELL2	-0,456814516
PPP4R2	-0,453401013
OSMR	-0,4528341
RBMXL1	-0,451007764
IPO11	-0,446892553
HLA-DRB1	-0,443305666
APOO	-0,441114061
SLC7A2	-0,439479497
ACO1	-0,436451409
GFPT2	-0,433871026
C10orf2	-0,433144275
ZNF468	-0,431075157
ZNF800	-0,430848277
FAM160A1	-0,425997848
SYT1	-0,42331469
AHI1	-0,423146325
PON2	-0,421558011
ETV3	-0,415675612
DUSP2	-0,415278167
ENGASE	-0,414411458
PUS1	-0,413404831
RALGAPA1	-0,413152716
FA2H	-0,41192704
EPHB2	-0,41111845
SULF2	-0,409920499
SLCO4A1	-0,409406537
FAT1	-0,408601735
CDC42EP2	-0,406915061
MT2A	-0,404896875
FAM83G	-0,404041756
DDHD1	-0,402872038
TNS3	-0,40272177
IL1RAP	-0,402271466
PLXNB1	-0,401876562
SLC25A37	-0,399083349
PDZD8	-0,397074126
MR1	-0,3966897
CHD7	-0,39667069
KLHL18	-0,396234726
TP53	-0,395679211
ZNF8	-0,395042357
ANKRD17	-0,394001015

SLC4A7	-0,391900109
LRP6	-0,391759311
MOCOS	-0,387334358
MBNL1	-0,386730323
ZFP36L2	-0,384912534
FAM206A	-0,383248295
SLC8B1	-0,382943572
APOBEC3F	-0,382146546
NUP155	-0,381365864
ZNF91	-0,380759376
CYLD	-0,38073688
FAM135A	-0,379513176
PRDM15	-0,378421689
PPAPDC2	-0,377486442
SPINT1	-0,377253739
FAM155B	-0,376949731
GCH1	-0,374070737
FAM86C2P	-0,373875736
PALB2	-0,372927689
SLC2A6	-0,372107953
IFNAR2	-0,37160798
APOBEC3C	-0,371596751
BID	-0,37103921
ZFC3H1	-0,370440069
POLR3B	-0,37030927
DDX21	-0,369115939
SNAPC4	-0,368819696
SUSD6	-0,368570375
PCCA	-0,368532486
ARHGAP26	-0,367358954
TRAF3IP2	-0,366949269
TRMT44	-0,366683031
ZNF281	-0,365998616
TOMM40L	-0,365360268
TMEM164	-0,365009876
NUDT16P1	-0,364155136
ABLIM1	-0,362094989
GTF3C4	-0,361609223
MYCBP2	-0,361421782
SOGA1	-0,361062482
LOC100130899	-0,358699995
SDC4	-0,357274135
DNAAF2	-0,35724813
WIPF3	-0,356193648
KIAA1804	-0,356045171
HS6ST2	-0,354388846
GPATCH2L	-0,353207394

TXLNG	-0,352423301
RAI14	-0,351660814
ZNF827	-0,351646661
NKX3-1	-0,350091162
CPNE7	-0,349927559
STEAP3	-0,346637479
PDPR	-0,346612267
PROCR	-0,345678121
WDR90	-0,345369625
CHST11	-0,344212298
MPZL2	-0,341140767
MPP6	-0,340905388
AHCTF1	-0,340506626
SCO2	-0,3403019
EPC2	-0,339871378
ZFAND4	-0,338541219
PNKD	-0,337826064
DHX34	-0,337821701
CSNK1A1	-0,336317037
TRAF3	-0,336276678
GDAP1	-0,334715826
SPTBN2	-0,333622541
MAP2K3	-0,332998196
PRMT9	-0,332154194
ICE1	-0,331546403
TRIB1	-0,3308704
ZNF271P	-0,32878075
WDR12	-0,328564332
PRKDC	-0,328531734
USP42	-0,326194035
RMND5A	-0,325639507
SBK1	-0,325284654
GTDC1	-0,324316846
CAPN15	-0,323365814
XPO4	-0,322928696
PSME4	-0,322373377
DPH2	-0,321849302
HPS5	-0,321171256
XBP1	-0,321116906
WDR43	-0,321065271
XXYLT1	-0,320879313
CEBPZ	-0,320155473
BMS1P20	-0,320031878
TMEM39A	-0,316268
TRIM36	-0,315626524
PLEKHH1	-0,315350641
B4GALNT3	-0,315112439

SEC23IP	-0,314737611
GUF1	-0,31463164
PTGES2	-0,31429799
TGS1	-0,313538567
OAT	-0,313379713
TBC1D30	-0,312838525
HMGCS1	-0,312612198
PTP4A2	-0,311383622
SLC31A1	-0,311325534
SIM2	-0,31032901
NOL11	-0,309673697
NEURL4	-0,309237452
TMEM125	-0,309056646
ABCA2	-0,308876867
PUM1	-0,308707293
USP6NL	-0,308495916
KIAA1147	-0,307594625
HEATR1	-0,3075512
ERCC6L2	-0,306337596
TEX10	-0,305450803
KCNQ2	-0,305279167
NOL6	-0,305157751
SLC25A12	-0,304670881
KIAA1143	-0,302268966
KLHL20	-0,302171774
TAF4B	-0,302018732
E2F4	-0,301472729
SLC18B1	-0,301431018
NSMAF	-0,301388549
BAZ1A	-0,300867318
LRPPRC	-0,30044914
PTPRK	-0,300206734
DNAJC16	-0,299624424
CYB5D1	-0,298952234
IARS	-0,29785681
NINJ1	-0,29776007
SUPV3L1	-0,297651519
METTL8	-0,297376734
ORC5	-0,297237858
DDX3X	-0,296763586
SLC25A32	-0,296680553
KAZN	-0,296608632
HCG18	-0,294077942
POLR3H	-0,293428685
CIPC	-0,292806075
PALD1	-0,291662033
ZNF286A	-0,291608469

RPS6KA3	-0,291058833
CMTM4	-0,289421951
TGIF1	-0,289023111
LETM1	-0,288706803
DENND2D	-0,287939559
RPS6KB1	-0,287842929
PPAT	-0,286767577
NAA15	-0,28635937
C6orf120	-0,286059151
CNOT1	-0,284817186
PLXNA1	-0,284808064
PRR14L	-0,284144025
SRC	-0,284041436
MRPL30	-0,283827797
WRAP73	-0,282404098
XPOT	-0,280833991
DNAJC2	-0,280221563
FBXL18	-0,279751873
ZNF142	-0,279320038
PCCB	-0,279041396
SQSTM1	-0,277940811
TPR	-0,277900622
NOP14	-0,276771118
CC2D2A	-0,274919598
RRP1B	-0,274471118
PDCD11	-0,273247936
NAT9	-0,272719422
MAK16	-0,272644024
EML4	-0,272195161
NKRF	-0,272067704
RNASEH1	-0,27169341
POP1	-0,271572491
NFE2L2	-0,271116743
DNM1L	-0,270855978
IFT57	-0,270807618
TRMT10C	-0,269416701
FAM120A	-0,268039472
THUMPD1	-0,26536383
CLDN12	-0,264878502
RIOK1	-0,263391443
ARRB1	-0,262252527
NFYA	-0,259629657
ZNF335	-0,258709276
ISG20L2	-0,258705122
ATP6V1C1	-0,258052263
SNRNP200	-0,258038019
GTF2I	-0,256876346

FAM98A	-0,256087319
AEN	-0,255807023
IDH3A	-0,255545716
PRMT5	-0,255525313
HSPA4	-0,254458819
RPL7L1	-0,253573223
TRMT5	-0,253432806
RABEP1	-0,252551367
KIAA0100	-0,251220047
PVRL1	-0,250336026
SKIV2L2	-0,249767848
PPRC1	-0,249168117
NUP98	-0,248245928
CDK12	-0,247836538
GCN1	-0,247121252
ACACA	-0,246561314
PRRC2C	-0,245643943
TRRAP	-0,243366437
ZNF384	-0,242514332
MINA	-0,242510194
KDM2B	-0,241584671
ESF1	-0,241505019
TTF2	-0,240954493
MED12	-0,240953382
INTS10	-0,240646647
RFT1	-0,240489139
KIAA0907	-0,240463069
ZNF207	-0,24009409
MAML1	-0,239811738
PPP6R2	-0,239222927
CLUH	-0,238729163
NUP153	-0,238598083
FADS1	-0,237944884
PRPF38B	-0,236968878
NOA1	-0,23661318
DHX30	-0,235020888
DENND5A	-0,234424778
EP400	-0,233585115
QSOX2	-0,233534683
WDR75	-0,232538022
NOLC1	-0,231941203
PFKM	-0,231897133
EIF3E	-0,23178662
MCCC2	-0,231786254
PRPF38A	-0,231522707
DEPDC5	-0,23028195
TOP1	-0,229436554

NEK9	-0,229062687
MOGS	-0,227405636
LARS	-0,224679693
YTHDF2	-0,224621639
VHL	-0,223514878
SPEN	-0,222104037
SLC41A1	-0,221653361
EIF2A	-0,221255886
SEC16A	-0,219134197
NUDT16	-0,217917723
MED1	-0,215350281
ZNF146	-0,215024643
TMEM120B	-0,21476491
TCERG1	-0,214422433
DCUN1D5	-0,214271773
NSD1	-0,214251746
MTIF2	-0,213816387
CACTIN	-0,212871105
COX15	-0,209850508
INTS1	-0,208177371
PMPCB	-0,207320619
GBA2	-0,205805555
KPNA6	-0,204017976
TSR1	-0,203931692
TRAF7	-0,203793274
ADCY3	-0,203318819
CAD	-0,20320761
PIEZO1	-0,202887886
ORC2	-0,200821537
ADGRL1	-0,197581006
POLR3A	-0,195978803
ETF1	-0,19513198
SLC7A6	-0,194737181
CPSF2	-0,192289017
SENP6	-0,191716702
SRCAP	-0,191135391
TCEB3	-0,190132229
GNL2	-0,183774324
PRPF8	-0,183203117
XRCC5	-0,182184653
ANAPC5	-0,1802782
BCLAF1	-0,169560591
LARP1	-0,154374671

Upregulated

Gene	log2FoldChange
KIF1C	0,15952036
ARPC5	0,16101527
CALM1	0,17156401
TBL2	0,18107747
PAQR4	0,18534271
PPP1R37	0,19547573
CALCOCO2	0,19712869
ATP6V1G1	0,1980781
GOLGA2	0,19964055
TMEM14C	0,2003145
IDH1	0,20046439
SUMF1	0,20103827
DEF6	0,2019171
ZFYVE21	0,20295959
NCK2	0,20398839
KBTBD2	0,21026326
NAGA	0,21066374
COPS8	0,21176983
TIPRL	0,21252858
DSTN	0,21308275
REEP5	0,21480672
PCMT1	0,21579416
PSMC2	0,22021877
CBLC	0,22117821
PLCD3	0,22125356
SLC35D2	0,22225822
ABRACL	0,22467295
TCIRG1	0,22556726
SUN1	0,22700549
PALM	0,22816202
TOX2	0,22874903
DMTN	0,2303921
SYPL1	0,23041942
HTATIP2	0,23205442
RHEB	0,23355304
ATP6V1E1	0,2340063
CRAT	0,23425758
SNX7	0,23429248
EID1	0,23539544
MICU1	0,23561681
SBDS	0,23788956
MAFK	0,2393126
SUN2	0,24006063
TRIM35	0,24081514
APLP1	0,24089697
ATP6AP2	0,240928

GPR161	0,24449211
SRD5A1	0,24473425
HEBP1	0,24537256
PPAP2C	0,24808549
RHOC	0,24865206
SLC50A1	0,25006034
KIAA1279	0,25082761
IQGAP3	0,25101894
FUNDC1	0,25103231
SPATS2L	0,25253272
MVP	0,25279646
CDK2	0,25325014
DERA	0,2538247
GS1-124K5,11	0,25495109
ETV5	0,25549728
GSDMD	0,25622801
GDI1	0,25670455
H3F3B	0,25800567
PREP	0,25810451
ISCA2	0,25940648
STYXL1	0,2602952
GNAI2	0,26059156
CTSZ	0,26277348
LASP1	0,2631714
COL6A1	0,26366074
ABHD12	0,26405764
POR	0,26470413
FAAH	0,26517553
MELK	0,26522841
HABP4	0,26598697
CETN2	0,26770171
CCNH	0,26982508
GHDC	0,26988863
TES	0,27285702
MAD2L1BP	0,27299246
ATP6AP1	0,27383609
SPRYD7	0,27407767
FHL3	0,27498728
CORO1C	0,2762877
TRIOBP	0,2771898
GSN	0,27877558
TPM4	0,27919779
OS9	0,2798409
MIDN	0,27997893
BNIP3	0,28021259
VGLL4	0,28057027
PHF1	0,28066595

AIG1	0,2810459
PHF13	0,28211109
CPNE2	0,2821931
MIF4GD	0,28392821
TNFRSF12A	0,28401674
PTGR1	0,28442679
CALM2	0,28477096
MXRA7	0,28480255
S100PBP	0,28510329
CAV2	0,2852008
CSPG4	0,28524906
EHD2	0,28580924
MAP3K3	0,28593399
GLIPR2	0,28643358
SHARPIN	0,28662291
SCOC	0,28671492
HSPB11	0,2887434
TNNT1	0,28890298
ADGRE5	0,29024874
ARL6IP1	0,2924957
ZDHHC12	0,29497532
DOK1	0,29509654
LMF2	0,29518844
FAM3C	0,2959252
TMEM134	0,29592729
ARL6IP5	0,2966659
PLEKHA2	0,29672529
IFRD1	0,29774767
CCDC92	0,29913658
UNC93B1	0,29930836
GAS2L1	0,30126224
AES	0,30148126
CERCAM	0,3022503
ANKRD9	0,30237347
TMEM87A	0,30392276
FBXO2	0,30460779
NXN	0,30519201
ARHGDIA	0,30535304
WSB1	0,30540757
NT5C	0,30557784
DCK	0,30658312
PCTP	0,30746473
SYNPO	0,30829294
CCDC9	0,30864316
DCBLD2	0,30931832
ZFP36	0,3104009
HSF2	0,31048974

GLUL	0,31173021
CNN3	0,31253959
SORBS3	0,31265236
OSBPL10	0,31304479
CRLF1	0,31351625
FN3KRP	0,31359267
ANXA2	0,31479804
ACTN4	0,3171093
CYTH2	0,31744373
TRIP10	0,31814185
HES2	0,31872148
CNEP1R1	0,31934752
MACF1	0,31972261
CD9	0,32059965
SMYD3	0,32199547
H1FX	0,3229483
ITPKB	0,32316255
KIAA1715	0,32374665
H1F0	0,32406778
HOXA10	0,32448072
ZNF185	0,32452379
BAIAP2	0,32482262
PLP2	0,32606024
CLK1	0,32633314
RASSF1	0,32735674
CTSV	0,32785123
WDR54	0,32915064
MYADM	0,33081652
CAPNS1	0,331184
SYTL1	0,33124792
CLUAP1	0,33126903
CRYZ	0,33195212
MYH9	0,33202391
CTDSPL	0,33338117
FAM89B	0,33338393
MSANTD3	0,33360112
DNAJB6	0,33391624
GGT7	0,33408222
ALS2	0,33529479
MFI2	0,33719395
RASSF7	0,33950524
C11orf68	0,33970738
IFI27L2	0,34167401
ZFYVE1	0,34193847
TM2D2	0,34298852
TMEM120A	0,34391004
SPSB1	0,34405944

FADD	0,34530697
AMOTL2	0,34559244
SLC25A4	0,34563232
EMP3	0,34614174
LATS2	0,34623846
INSIG2	0,34722408
TCF7	0,34732893
CENPU	0,34840889
RGS3	0,34845512
UBXN2A	0,35287472
DLX4	0,35393376
FAM64A	0,35438571
WRB	0,35442345
RBCK1	0,35490778
KATNAL1	0,35695033
OLFML2A	0,35709596
POLD4	0,35720489
DRAP1	0,35892321
REEP1	0,36005918
ALDH3B1	0,36068569
ING1	0,36072851
EBF4	0,36113711
CCDC88B	0,36114638
ZNF250	0,36258159
KALRN	0,36341782
LZTS1	0,36531075
GPC4	0,36662669
ZNF365	0,36717927
LMBRD1	0,3672833
RASSF4	0,36793712
HAUS8	0,37111786
ZNRF2	0,37112264
PECR	0,37121442
FRS3	0,37254546
CALB2	0,3726836
SOX9	0,37352369
PRKAG2	0,37397597
ENDOD1	0,37399651
C5orf30	0,37435216
LMNA	0,37505298
ROGDI	0,37743325
C4orf33	0,37774752
MROH6	0,37784813
FUT8	0,37805882
PRDX5	0,37887438
CEP55	0,38032442
IRF9	0,38143056

HOXC6	0,38222148
ACAP3	0,38455146
TMEM65	0,38466186
MSLN	0,38525952
NES	0,38628126
ZNF385A	0,38675847
RAD54L	0,38705304
FSCN1	0,38714627
CTNNAL1	0,38755453
PRR7	0,38872707
PAAF1	0,38982985
IER5L	0,38999353
CCDC85B	0,39016509
EFNA2	0,39126996
HOXB7	0,39227527
LSR	0,39310209
RIPK4	0,39380027
MAP3K14	0,39470868
SRD5A3	0,39508166
RGS14	0,39551022
CASP6	0,39551104
RAB20	0,39557372
NPC2	0,39605715
SHISA4	0,39647541
ANXA1	0,40113203
PLA2G15	0,40172117
STMN1	0,40243174
CRIP1	0,40280202
PPDPF	0,404133
MFGE8	0,40549256
ARC	0,40588672
ENO2	0,40827839
SAP30	0,4086073
FLOT1	0,40952479
PMP22	0,40965073
TAB3	0,41584802
FXYD5	0,41884026
TNS2	0,42049609
RAMP1	0,42065132
HOXB5	0,42072733
HES1	0,42233178
NUCB2	0,42278995
STK17A	0,42283623
CUL4B	0,42456913
KNDC1	0,42461253
PTTG1IP	0,42488865
SEPT4	0,42621823

SLCO1B3	0,42734224
CRIP2	0,42804125
RNF122	0,42836869
ALCAM	0,42862085
C8orf58	0,42876038
UPK1A-AS1	0,42883487
COL13A1	0,42903329
ASAP2	0,43059437
LOXL2	0,43137861
SARDH	0,4320649
TNFSF12	0,43326189
AXL	0,43326419
MOK	0,43351723
LIPE	0,43500663
NKX6-1	0,43798315
TMEM59L	0,43943347
LINC00152	0,44003815
CDH15	0,44046713
ABLIM3	0,44367416
TMEM173	0,44448033
TLE6	0,44485175
LHX6	0,44609143
CACFD1	0,44648444
DBP	0,45015809
DUSP23	0,45097263
CHRD	0,45226168
MGC50722	0,45243131
WNT6	0,45248785
CYB5R2	0,45285456
STIM1	0,45951865
GATA2	0,45972038
LAMB1	0,45996662
PRICKLE1	0,46489738
CDC42EP3	0,46497099
PLK2	0,46613526
REEP3	0,46832531
MXD3	0,47311293
UBASH3B	0,47867429
HEXIM2	0,47906722
ADAMTS14	0,48112264
KLF2	0,48494619
DCAF4	0,4860126
WWC3	0,48913835
ULBP2	0,49145855
ADM	0,49378013
C11orf71	0,49454281
HTR1D	0,49868936

CLIC3	0,49972636
CDKN2C	0,49983164
SLC22A18AS	0,50370007
ESAM	0,50794729
S100A10	0,50850248
MAP2	0,50971426
TBX3	0,5111496
CD22	0,51136187
GLT8D2	0,51172413
ADAMTS17	0,51359956
ST6GALNAC2	0,51404687
PDLIM2	0,51669116
ADSSL1	0,51682065
GCA	0,51795017
ETS1	0,51818656
ATP8B3	0,52447061
PTPRN2	0,52503258
MBNL3	0,52816228
NKX2-8	0,52853886
MPND	0,53021747
SAMD11	0,53252751
PRIMA1	0,5327471
RNASE4	0,53556006
PADI2	0,53799233
MALL	0,54319386
FOLR1	0,54985265
CYP26B1	0,54985336
TENM3	0,55236833
FNDC4	0,55496682
TUBB4A	0,5563716
SNORA32	0,56035202
ST6GALNAC5	0,56343317
PPM1K	0,56346184
ZFYVE28	0,56906008
FAM83E	0,57041368
UBALD2	0,57283219
MEF2C	0,5730158
SP5	0,57720215
TLN2	0,57761569
CLIP4	0,58030926
MIR22HG	0,58473256
ANKRD37	0,58488108
ITGB4	0,58723589
PLCB2	0,58734691
FBXO32	0,59449173
OTUD1	0,59488679
F10	0,59880904

FOXD1	0,60283809
SMPD1	0,6078954
LCAT	0,6166614
STRA6	0,62510892
CD24	0,6267846
SESN3	0,63016574
KCNIP3	0,63571255
CYP4F11	0,63588216
ZFP30	0,63770364
SP110	0,64107287
ARL4C	0,6418952
BVES	0,64298943
LINC00511	0,64709172
HSD11B1L	0,65089314
KHDRBS3	0,65198979
CTGF	0,65774088
EFNA5	0,66424044
WNT16	0,66710382
FAM189A2	0,67148984
GPR162	0,6728861
FRY	0,68071472
AAMDC	0,70598605
GNG13	0,70677734
ENC1	0,71022217
ART5	0,71361136
HEG1	0,71841194
RGS9	0,72219678
PHYHIP	0,72236538
HMCN1	0,72987865
LYPD1	0,73282266
MMP28	0,74321357
SHC2	0,74623984
JMJD1C-AS1	0,76734714
MAP3K15	0,771474
REEP2	0,77462948
NPIPA5	0,7826008
MAP1B	0,789897
EVI2B	0,79432141
INHBB	0,79722466
SNAI2	0,80586097
LOC102724279	0,81550344
ALPP	0,81896411
B3GALT4	0,82046565
DLK2	0,83237643
GPR176	0,84959781
FBP1	0,8742353
LOC101927884	0,88024482

PKIB	0,88033221
HPD	0,88146616
GUCY2D	0,89684466
PADI1	0,91280084
SLC38A5	0,95677258
ASCL5	0,96303998
GNRH1	0,97049317
KRT81	0,97601073
GRIK2	0,99224553
CBR3	1,0465E+13
LCTL	1,1434E+13
TGFBI	1,5947E+13
CDK15	1,6705E+13
FEZF1	1,0144E+14
YBX2	1,0193E+14
GPER1	1,026E+14
CGB2	1,0324E+14
KRT9	1,055E+14
KIAA1549L	1,0604E+14
PGM5P2	1,0763E+14
PTHLH	1,0835E+14
GSN-AS1	1,0874E+14
KRT86	1,1151E+14
OVOL2	1,1282E+14
SIX2	1,2134E+14
PI16	1,2683E+14
ID3	1,3124E+14
ACTA1	1,3426E+14
PIR	1,3656E+14
LINC01605	1,3764E+14
TBC1D3F	1,4323E+14
SERTAD4	1,5595E+14
CXCR3	1,7696E+14
KANSL1-AS1	1,9799E+14
FOXS1	2,6719E+14
RPH3A	2,7468E+14
SLCO2A1	3,4889E+14
