# Supplementary material for: The synergism of SMC1A cohesin gene silencing and bevacizumab against colorectal cancer
Source: J Exp Clin Cancer Res. 2024 Feb 16;43:49. doi: 10.1186/s13046-024-02976-2 (PMC10870497; doi:10.1186/s13046-024-02976-2)
Supplement: Supplementary file 13 — Additional file 13: Table S8. Dysregulated pathways following shRNA, bevacizumab and combo treatments. [file 13046_2024_2976_MOESM13_ESM.pdf]

Table S8. Dysregulated pathways following shRNA, bevacizumab and combo treatments.

**shRNA**	Number of genes
U2-type prespliceosome assembly (GO:1903241)	5
peptidyl-threonine dephosphorylation (GO:0035970)	5
ribosomal large subunit biogenesis (GO:0042273)	5
chaperone mediated protein folding requiring cofactor (GO:0051085)	5
protein acetylation (GO:0006473)	4
inactivation of MAPK activity (GO:0000188)	4
negative regulation of centriole replication (GO:0046600)	3
7-methylguanosine cap hypermethylation (GO:0036261)	3

**Bevacizumab**	Number of genes
response to lipopolysaccharide (GO:0032496)	12
negative regulation of cell growth (GO:0030308)	11
regulation of actin cytoskeleton organization (GO:0032956)	9
actin cytoskeleton reorganization (GO:0031532)	8
neuron apoptotic process (GO:0051402)	8
response to nutrient (GO:0007584)	8
positive regulation of substrate adhesion-dependent cell spreading (GO:1900026)	7
release of sequestered calcium ion into cytosol (GO:0051209)	6
endoplasmic reticulum organization (GO:0007029)	6
platelet aggregation (GO:0070527)	6
glutathione metabolic process (GO:0006749)	6
T cell chemotaxis (GO:0010818)	5
positive regulation of innate immune response (GO:0045089)	5
positive regulation of mitotic cell cycle (GO:0045931)	5
myelin assembly (GO:0032288)	4
regulation of high voltage-gated calcium channel activity (GO:1901841)	4
negative regulation of ryanodine-sensitive calcium-release channel activity (GO:0060315)	4
vitamin D receptor signaling pathway (GO:0070561)	3
negative regulation of toll-like receptor 2 signaling pathway (GO:0034136)	3

**Combo**	Number of genes
defense response to virus (GO:0051607)	19
positive regulation of angiogenesis (GO:0045766)	18
actin cytoskeleton organization (GO:0030036)	18
positive regulation of I-kappaB kinase/NF-kappaB signaling (GO:0043123)	18
positive regulation of protein phosphorylation (GO:0001934)	18
skeletal system development (GO:0001501)	15
DNA-templated transcription, elongation (GO:0006354)	15
cellular response to tumor necrosis factor (GO:0071356)	14
canonical Wnt signaling pathway (GO:0060070)	13
negative regulation of cell growth (GO:0030308)	13
rRNA processing (GO:0006364)	13
cellular response to mechanical stimulus (GO:0071260)	12

transcription, DNA-templated (GO:0006351)	12
cellular response to interleukin-1 (GO:0071347)	11
positive regulation of cell growth (GO:0030307)	11
wound healing (GO:0042060)	11
negative regulation of cysteine-type endopeptidase activity involved in apoptotic process (GO:0043154)	10
response to wounding (GO:0009611)	10
neural tube closure (GO:0001843)	10
mRNA transport (GO:0051028)	10
GO:0030199~collagen fibril organization	9
heart looping (GO:0001947)	9
cellular response to drug (GO:0035690)	9
G1/S transition of mitotic cell cycle (GO:0000082)	9
positive regulation of intrinsic apoptotic signaling pathway (GO:2001244)	8
regulation of neurogenesis (GO:0050767)	8
ephrin receptor signaling pathway (GO:0048013)	8
protein kinase B signaling (GO:0043491)	8
positive regulation of fat cell differentiation (GO:0045600)	8
positive regulation of extrinsic apoptotic signaling pathway (GO:2001238)	7
positive regulation of release of cytochrome c from mitochondria (GO:0090200)	7
T cell differentiation in thymus (GO:0033077)	7
positive regulation of Wnt signaling pathway (GO:0030177)	7
cellular response to peptide hormone stimulus (GO:0071375)	6
hematopoietic stem cell differentiation (GO:0060218)	6
motile cilium assembly (GO:0044458)	6
establishment of skin barrier (GO:0061436)	6
positive regulation of cell migration involved in sprouting angiogenesis (GO:0090050)	6
mitochondrial calcium ion transport (GO:0006851)	5
response to steroid hormone (GO:0048545)	5
post-anal tail morphogenesis (GO:0036342)	5
negative regulation of stem cell differentiation (GO:2000737)	5
nuclear envelope organization (GO:0006998)	5
female genitalia development (GO:0030540)	4
osteoclast development (GO:0036035)	4
positive regulation of transcription from RNA polymerase II promoter in response to stress (GO:0036003)	4
positive regulation of rRNA processing (GO:2000234)	4
glomerular basement membrane development (GO:0032836)	4
nuclear-transcribed mRNA catabolic process, deadenylation-dependent decay (GO:0000288)	4
positive regulation of phagocytosis, engulfment (GO:0060100)	4
oxidative stress-induced premature senescence (GO:0090403)	3
response to gonadotropin (GO:0034698)	3
epithelium development (GO:0060429)	3
Wnt signaling pathway involved in somitogenesis (GO:0090244)	3
